# pH-switchable nanozyme cascade catalysis: a strategy for spatial–temporal modulation of pathological wound microenvironment to rescue stalled healing in diabetic ulcer

**DOI:** 10.1186/s12951-021-01215-6

**Published:** 2022-01-04

**Authors:** Xuancheng Du, Bingqing Jia, Weijie Wang, Chengmei Zhang, Xiangdong Liu, Yuanyuan Qu, Mingwen Zhao, Weifeng Li, Yanmei Yang, Yong-Qiang Li

**Affiliations:** 1 Institute of Advanced Interdisciplinary Science, School of Physics, Shandong University, Jinan, 250100 China; 2 Laboratory Animal Center of Shandong University, Jinan, 250012 China; 3 College of Chemistry, Chemical Engineering and Materials Science, Collaborative Innovation Center of Functionalized Probes for Chemical Imaging in Universities of Shandong, Key Laboratory of Molecular and Nano Probes, Ministry of Education, Shandong Normal University, Jinan, 250014 China; 4 Suzhou Research Institute, Shandong University, Suzhou, 215123 China

**Keywords:** Biofilm eradication, Cascade catalysis, Diabetic ulcer, Nanozyme, Pathological microenvironment modulation

## Abstract

The management of diabetic ulcer (DU) to rescue stalled wound healing remains a paramount clinical challenge due to the spatially and temporally coupled pathological wound microenvironment that features hyperglycemia, biofilm infection, hypoxia and excessive oxidative stress. Here we present a pH-switchable nanozyme cascade catalysis (PNCC) strategy for spatial–temporal modulation of pathological wound microenvironment to rescue stalled healing in DU. The PNCC is demonstrated by employing the nanozyme of clinically approved iron oxide nanoparticles coated with a shell of glucose oxidase (Fe_3_O_4_-GOx). The Fe_3_O_4_-GOx possesses intrinsic glucose oxidase (GOx), catalase (CAT) and peroxidase (POD)-like activities, and can catalyze pH-switchable glucose-initiated GOx/POD and GOx/CAT cascade reaction in acidic and neutral environment, respectively. Specifically, the GOx/POD cascade reaction generating consecutive fluxes of toxic hydroxyl radical spatially targets the acidic biofilm (pH ~ 5.5), and eradicates biofilm to shorten the inflammatory phase and initiate normal wound healing processes. Furthermore, the GOx/CAT cascade reaction producing consecutive fluxes of oxygen spatially targets the neutral wound tissue, and accelerates the proliferation and remodeling phases of wound healing by addressing the issues of hyperglycemia, hypoxia, and excessive oxidative stress. The shortened inflammatory phase temporally coupled with accelerated proliferation and remodeling phases significantly speed up the normal orchestrated wound-healing cascades. Remarkably, this Fe_3_O_4_-GOx-instructed spatial–temporal remodeling of DU microenvironment enables complete re-epithelialization of biofilm-infected wound in diabetic mice within 15 days while minimizing toxicity to normal tissues, exerting great transformation potential in clinical DU management. The proposed PNCC concept offers a new perspective for complex pathological microenvironment remodeling, and may provide a powerful modality for the treatment of microenvironment-associated diseases.

**Supplementary Information:**

The online version contains supplementary material available at 10.1186/s12951-021-01215-6.

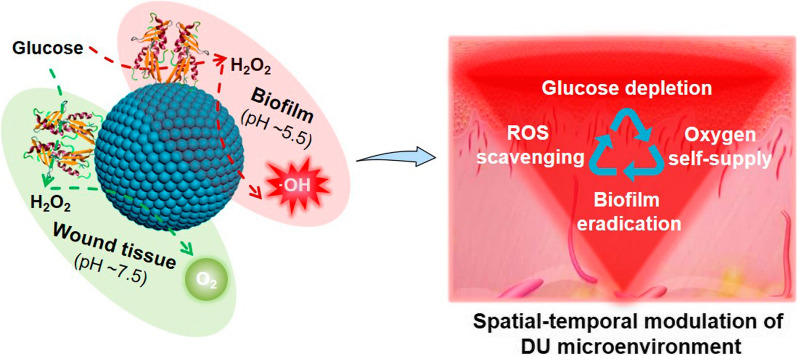

## Introduction

Diabetes affecting 451 million people has become a global epidemic, and poses mounting public health concerns due to its clinical complications [1]. Diabetic ulcer (DU) characterized by extremely slow or even stagnant wound-healing cascades (hemostasis, inflammation, proliferation and remodeling) that usually form on the feet and legs, is an intractable complication of diabetes [2]. Despite continuous improvements in tissue engineering and regeneration, the treatment of DU remains a grand clinical challenge, resulting in high rate of limb amputations and causing an enormous medical and financial burden [3, 4]. The spatially and temporally coupled pathological wound microenvironment that features hyperglycemia, hypoxia, biofilm infection and excessive oxidative stress, is the culprit for the dilemma of DU treatment [5]. Specifically, the spatially coexisted biofilm (bacterial community wrapped by self-produced extracellular polymeric substances) and wound tissue have completely different pathological characteristics, setting a myriad of stumbling blocks and greatly enhancing the difficulty of DU [6, 7]. Meanwhile, the prolonged inflammatory phase resulted from biofilm infection is temporally coupled with the stagnant proliferation and remodeling phases attributed to the synergy of hyperglycemia, hypoxia, and excessive oxidative stress in DU [8–10], causing non-healable wounds. Therefore, reshaping the spatially and temporally coupled pathological wound microenvironment to rescue stalled healing is critical for the treatment of DU.

By individually targeting the issues of hyperglycemia, hypoxia, bacterial infection and excessive oxidative stress, sophisticated strategies for pathological wound microenvironment remodeling have been presented including glucose regulation [11], local oxygen delivery [12, 13], photothermal and photodynamic antimicrobial therapy [14, 15], and ROS scavenging [16–18]. A variety of effective multifunctional biomaterials such as hydrogels and electrospun polymer micro/nanofibers, have been prepared based on these strategies to alleviate pathological microenvironment and help restore the healing cascades in chronic wound [19–22]. However, their therapeutic effect on DU is not ideal due to the mutual influence and synergy of hyperglycemia, hypoxia, biofilm infection, and excessive oxidative stress in diabetic wound [5]. To simultaneously address all these DU-related issues, recently integrated hydrogel systems have been reported by assembling function modules of glucose depletion, oxygen delivery, bacterial biofilm elimination and ROS removal into one hydrogel, notably accelerating the healing of diabetic wound [6, 23, 24]. However, these function modules of the integrated hydrogels are independent of each other and lack the spatial–temporal synergy mechanism to achieve better wound healing effects. Moreover, this all-in-one assembly strategy makes the composition of the integrated hydrogels extremely complicated, and the potential immunogenicity and biotoxicity of these hydrogel components cause substantial anxiety, greatly restricting their clinical applications [25]. Therefore, alternative strategy to prepare biocompatible microenvironment modulator with simple composition capable of spatially and temporally addressing the four DU microenvironment-related issues is highly desirable in DU management.

Research into enzyme cascade catalysis may provide insight into potential strategies for the development of desired biocompatible DU microenvironment modulator. Enzymes are natural biomaterials capable of catalyzing various chemical reactions to mediate biological processes [26]. In living organisms, enzyme cascade systems have been evolved by confining cooperating enzymes within compartments to boost their catalytic performances without the separation of intermediates for spatial–temporal modulation of complex physiological microenvironment [27, 28]. Inspired by this, enzyme-medicated cascade catalysis strategy could be developed for DU microenvironment modulation. However, due to the limitation of the specificity of enzyme-catalyzed reactions as well as high cost and harsh usage condition [29], enzyme cascade catalysis systems with simple composition that can concurrently address the four DU-related issues remain elusive. Recently, investigation on nanozyme has demonstrated that some nanomaterials (e.g., metal-oxide and carbon-based nanoparticles) possess distinctive pathological conditions-switchable multiple enzyme-like activities, indicating the feasibility of employing one nanozyme to catalyze cascade reactions [30–33]. Therefore, we hypothesize that by combing the concept of enzyme cascade catalysis and nanozyme in the design of pathological wound microenvironment spatial–temporal modulator, a robust strategy for DU management could be developed.

**Scheme 1:**
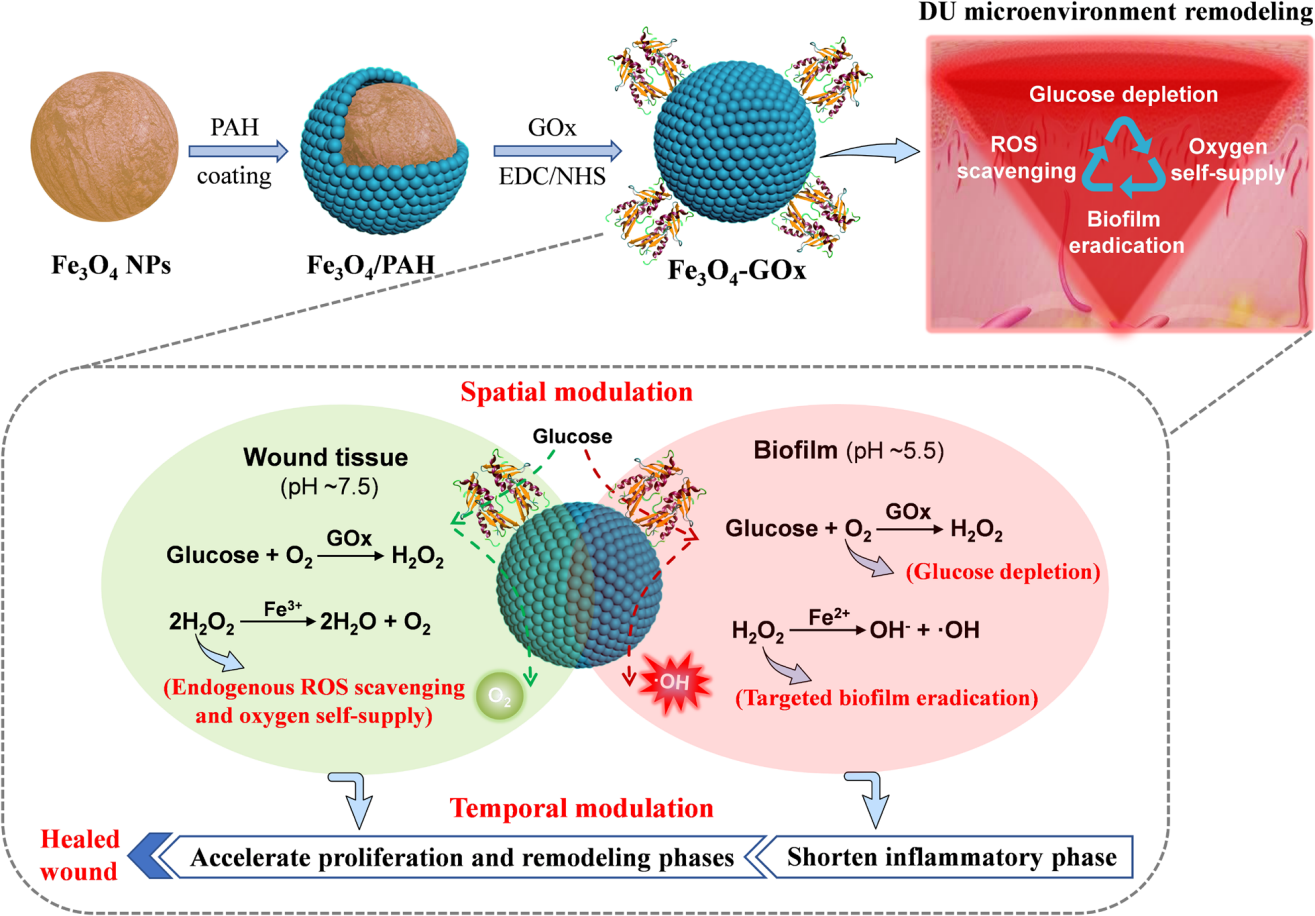
Conceptual illustration of the Fe_3_O_4_-GOx-instructed PNCC strategy for spatial–temporal modulation of pathological wound microenvironment to rescue stalled healing in DU

Herein, we introduce a pH-switchable nanozyme cascade catalysis (PNCC) strategy for spatial–temporal modulation of pathological wound microenvironment to rescue stalled healing in DU (Scheme 1). The nanozyme we designed (named Fe_3_O_4_-GOx) is composed of an iron oxide nanoparticle (Fe_3_O_4_ NPs) core and a glucose oxidase (GOx) shell. GOx is an oxidoreductase that can catalyze the oxidization of glucose to produce hydrogen peroxide (H_2_O_2_) [34]. Fe_3_O_4_ NPs approved by the United States Food and Drug Administration (FDA), exhibits intrinsic pH-dependent peroxidase (POD) and catalase (CAT)-like activities that can catalyze the decomposition of H_2_O_2_ into oxygen and hydroxyl radicals (·OH), respectively [35–37]. Therefore, pH-switchable GOx/POD and GOx/CAT cascade reactions can be carried out in the Fe_3_O_4_-GOx with closely co-localized GOx and Fe_3_O_4_ NPs. Specifically, the GOx/POD cascade reaction generating consecutive fluxes of toxic hydroxyl radical spatially targets the acidic biofilm (pH ~ 5.5), and eradicates biofilm to shorten the inflammatory phase and initiate the normal wound healing of proliferation and remodeling. Furthermore, the GOx/CAT cascade reaction producing consecutive fluxes of oxygen spatially targets the neutral wound tissue, and accelerates the proliferation and remodeling phases of wound healing by addressing the issues of hyperglycemia, hypoxia, and excessive oxidative stress. The shortened inflammatory phase temporally coupled with accelerated proliferation and remodeling phases significantly speed up the normal orchestrated wound-healing cascades, enabling efficient DU treatment.

## Results and discussion

### Preparation and characterization of Fe_3_O_4_-GOx

**Fig. 1:**
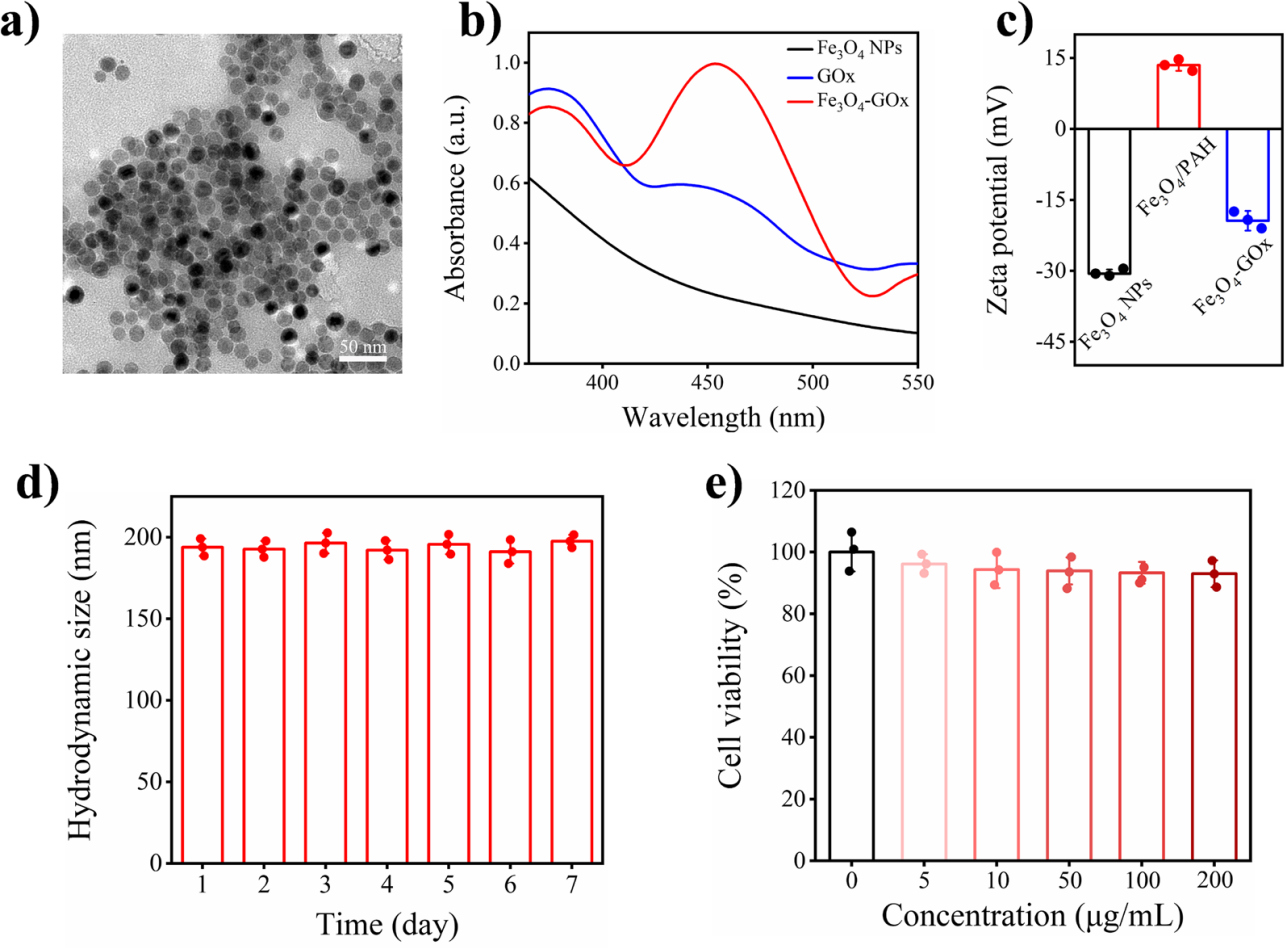
Characterization of Fe_3_O_4_-GOx. **a** TEM image of Fe_3_O_4_-GOx nanozyme. **b** UV-vis absorption spectra of Fe_3_O_4_ NPs, GOx, and Fe_3_O_4_-GOx nanozyme. **c** Zeta potentials of Fe_3_O_4_ NPs, PAH-coated Fe_3_O_4_ NPs (Fe_3_O_4_/PAH), and Fe_3_O_4_-GOx nanozyme in DI water. **d** Hydrodynamic diameter of Fe_3_O_4_-GOx nanozyme in PBS buffer (0.01 M, pH 7.4) during 7 days of storage. **e** Viability of HUVEC cells after incubation with Fe_3_O_4_-GOx nanozyme at various concentrations of iron element for 24 h. In **c**–**e**, the values of zeta potential, hydrodynamic diameter and cell viability represent the mean of three independent experiments, and the error bars indicate the standard deviation (SD) from the mean

In typical experiments, magnetic Fe_3_O_4_ NPs was first synthesized based on a thermal decomposition method (Additional file 1: Figs. S1 and S2) [38, 39], and Fe_3_O_4_-GOx nanozyme was then prepared by poly (allylamine hydrochloride) (PAH) electrostatic coating and GOx covalent modification (Scheme 1). The transmission electron microscopy (TEM) image shows that the prepared Fe_3_O_4_-GOx nanozyme had a homogeneous and well-defined spherical structure with an average size of 12.7 ± 2.5 nm (Fig. 1a). The UV-vis absorption spectra show that the Fe_3_O_4_-GOx nanozyme exhibited the characteristic absorption peaks of GOx at around 377 and 455 nm respectively, confirming the successful conjugation of GOx molecules (Fig. 1b). The loading amount of GOx on Fe_3_O_4_-GOx nanozyme (1.5 ± 0.2 mg of GOx per mg of iron element) was quantitatively determined by bicinchoninic acid (BCA) assay (Additional file 1: Fig. S3) [40]. In addition, the whole preparation process of Fe_3_O_4_-GOx nanozyme could be easily monitored and confirmed by the results of reversed zeta potential (Fig. 1c) and increased hydrodynamic size (Additional file 1: Fig. S4). Moreover, no significant increase of hydrodynamic size was found for Fe_3_O_4_-GOx nanozyme during long-term storage in PBS buffer at different temperatures (4 and 25 °C), revealing the high structural stability and excellent solubility of Fe_3_O_4_-GOx in an aqueous environment (Fig. 1d and Additional file 1: Fig. S5). Furthermore, from the methyl thiazolyl tetrazolium (MTT) assay, it was found that the human umbilical vein endothelial cells (HUVEC) possessed high viability (≥ 93%) after incubation with Fe_3_O_4_-GOx nanozyme at various concentrations, demonstrating the outstanding in vitro biocompatibility of Fe_3_O_4_-GOx nanozyme (Fig. 1e). This excellent in vivo biocompatibility is reasonable by considering the low biotoxicity of GOx and clinically approved Fe_3_O_4_ NPs [34, 41].

### Neutral pH-switchable GOx/CAT cascade catalysis of Fe_3_O_4_-GOx

**Fig. 2:**
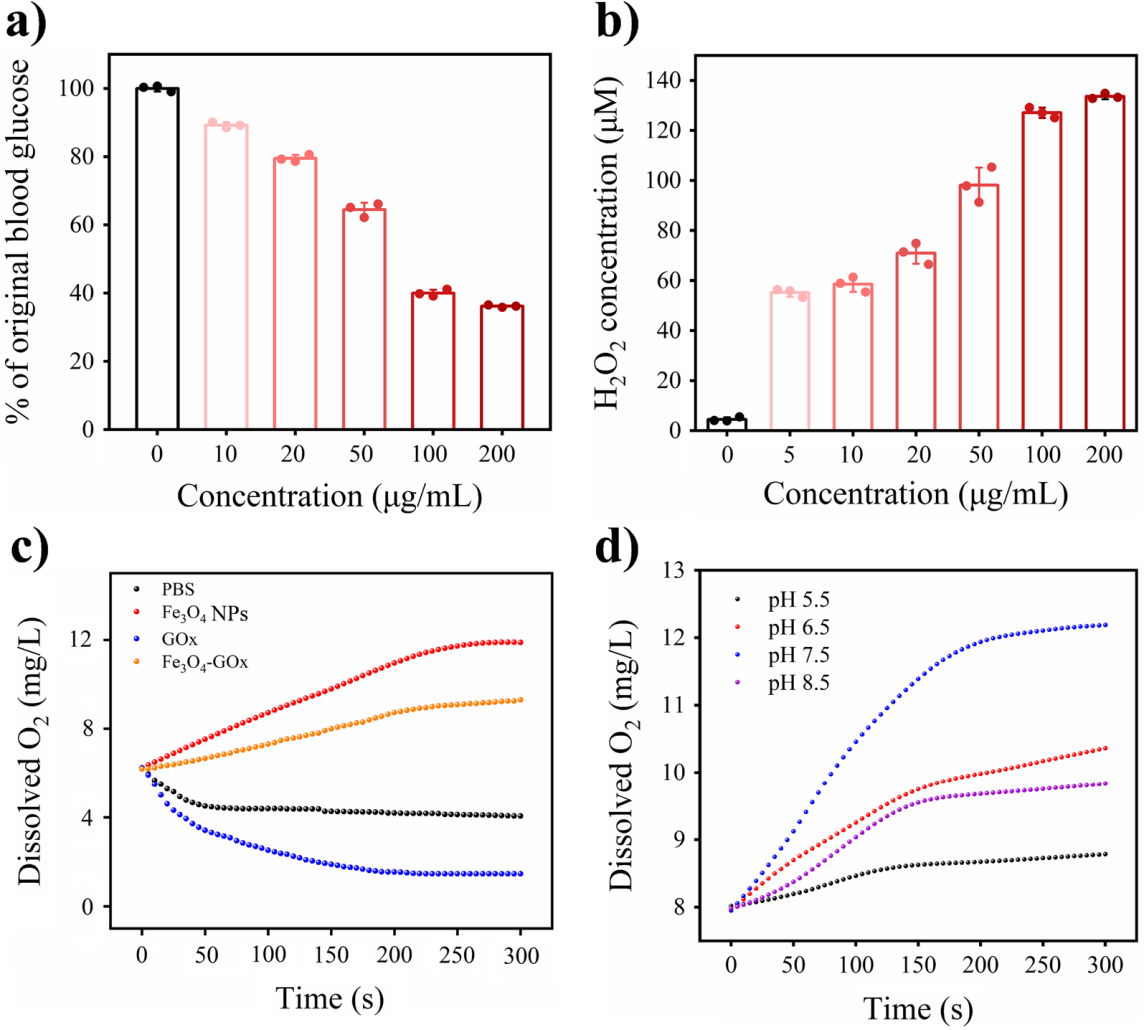
Neutral pH-switchable GOx/CAT cascade catalysis capability of Fe_3_O_4_-GOx. **a** The depletion of glucose in diabetic blood sample (containing around 20 mM of glucose) after incubation with different concentrations of Fe_3_O_4_-GOx nanozyme for 5 min. **b** The concentration of H_2_O_2_ generated in glucose solution (20 mM, pH 7.5) after incubation with different concentrations of Fe_3_O_4_-GOx nanozyme for 5 min. **c** The change of dissolved O_2_ concentration in the mixed solution of glucose (20 mM) and H_2_O_2_ (7 mM) in pH 7.4 after incubation with PBS, Fe_3_O_4_ NPs (200 µg/mL of iron element), GOx (300 µg/mL) and Fe_3_O_4_-GOx (200 µg/mL of iron element) for 5 min, respectively. **d** The change of dissolved O_2_ concentration in the solution of glucose (20 mM) and H_2_O_2_ (10 mM) with different pH values after incubation with Fe_3_O_4_-GOx (200 µg/mL of iron element) for 5 min. In **a** and **b**, the values of glucose consumption (% of original blood glucose) and H_2_O_2_ concentration represent the mean of three independent experiments, and the error bars indicate the SD from the mean

By assembling GOx with Fe_3_O_4_ NPs, the Fe_3_O_4_-GOx is expected to acquire the catalytic activities of GOx, CAT and POD, and can elicit coupled GOx/CAT and GOx/POD cascade reactions. To confirm this expectation, the GOx activity of Fe_3_O_4_-GOx to realize glucose depletion was first investigated. As shown in Fig. 2a, the glucose concentration of diabetic blood sample significantly decreased with the increase of Fe_3_O_4_-GOx nanozyme (blood glucose concentration was reduced by 62% after incubation with 200 µg/mL of Fe_3_O_4_-GOx for 5 min), indicating the depletion of glucose. Meanwhile, H_2_O_2_ was detected in the glucose solution after incubation with Fe_3_O_4_-GOx nanozyme, and its concentration was found to be gradually increased with the increase of Fe_3_O_4_-GOx nanozyme (Fig. 2b), demonstrating the oxidation of glucose in the presence of Fe_3_O_4_-GOx nanozyme. The results of glucose depletion and H_2_O_2_ production together prove the GOx activity of Fe_3_O_4_-GOx nanozyme. Additional file 1: Fig. S6 shows the production of H_2_O_2_ in glucose solution after incubation with Fe_3_O_4_-GOx nanozyme for different times. It was found that the concentration of H_2_O_2_ gradually decreased with the extension of glucose and Fe_3_O_4_-GOx incubation time, indicating the decomposition of H_2_O_2_ produced in glucose oxidation. This phenomenon may be ascribed to the occurrence of GOx/CAT and GOx/POD cascade reactions in the Fe_3_O_4_-GOx/glucose system, due to the CAT and POD activities of Fe_3_O_4_-GOx nanozyme to catalyze the decomposition of H_2_O_2_. The co-existent POD and CAT-like activities of Fe_3_O_4_-GOx was attributed to the mixed valence of Fe^2+^ and Fe^3+^, as evidenced by the result of X-ray photoelectron spectroscopy (XPS) analysis (Additional file 1: Fig. S7).

To assess the CAT activity of Fe_3_O_4_-GOx and confirm the occurrence of coupled GOx/CAT cascade reaction in the Fe_3_O_4_-GOx/glucose system, Fe_3_O_4_-GOx nanozyme was incubated with the mixture of glucose and H_2_O_2_ (glucose-H_2_O_2_), and the production of O_2_ was evaluated. As shown in Fig. 2c, considerable level of O_2_ was detected in the systems of Fe_3_O_4_-GOx/glucose-H_2_O_2_ and Fe_3_O_4_ NPs/glucose-H_2_O_2_ rather than the control systems of PBS/glucose-H_2_O_2_ and GOx/glucose-H_2_O_2_, indicating the remarkable CAT activity of Fe_3_O_4_-GOx and Fe_3_O_4_ NPs. Moreover, compared to the system of Fe_3_O_4_ NPs/glucose-H_2_O_2_, a relatively lower O_2_ concentration was found in the system of Fe_3_O_4_-GOx/glucose-H_2_O_2_ due to the O_2_ consumption during glucose oxidation, confirming the occurrence of coupled GOx/CAT cascade reaction in the system of Fe_3_O_4_-GOx/glucose-H_2_O_2_. This result indicates that the toxic H_2_O_2_ produced in glucose oxidation as well as oxidative stress progression of DU wound can be converted into beneficial O_2_ by Fe_3_O_4_-GOx, making synergistic tissue hypoxia and oxidative stress amelioration in DU become possible and forming a self-oxygen supply system to accelerate glucose oxidation. In addition, the production of O_2_ in the Fe_3_O_4_-GOx/glucose-H_2_O_2_ systems with different pH values was assessed. As shown in Fig. 2d, the system of Fe_3_O_4_-GOx/glucose-H_2_O_2_ under neutral (pH 7.5) and slightly acidic (pH 6.5) conditions produced much higher level of O_2_ compared to that under acidic (pH 5.5) and alkaline (pH 8.5) conditions, showing a neutral environment-preferred GOx/CAT cascade reaction. This pH-dependent CAT activity of Fe_3_O_4_-GOx nanozyme is consistent with the Fe_3_O_4_ NPs previously reported [36]. The oxidation process of glucose catalyzed by GOx is accompanied by the formation of gluconic acid, which would decrease the pH of the surrounding microenvironment and further affect the CAT-like activity of Fe_3_O_4_-GOx. Additional file 1: Fig. S8 shows the pH change of the Fe_3_O_4_-GOx/glucose system. It was found that the gluconic acid generated basically did not affect the pH value of the Fe_3_O_4_-GOx/glucose system (pH only dropped by 0.32 ± 0.14 within 60 min) at the glucose working concentration (20 mM) we employed. Predictably, such a small pH change will not affect the CAT-like activity as well as the GOx/CAT cascade catalysis performance of Fe_3_O_4_-GOx/glucose system.

### Acidic pH-switchable GOx/POD cascade catalysis of Fe_3_O_4_-GOx

**Fig. 3:**
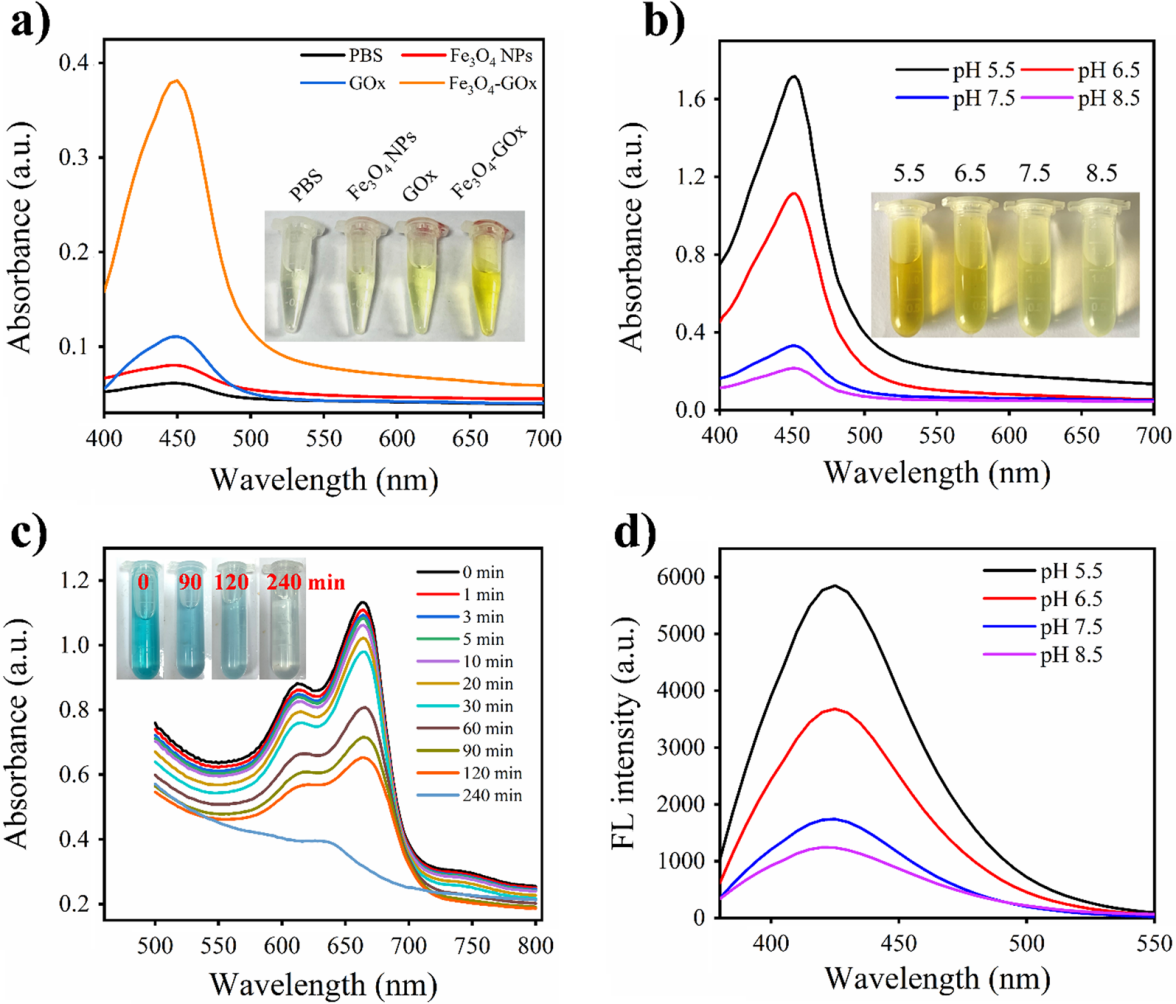
Acidic pH-switchable GOx/POD cascade catalysis capability of Fe_3_O_4_-GOx. **a** UV-vis absorption spectra of glucose solution (20 mM, pH 5.5) after incubation with PBS, Fe_3_O_4_ NPs (200 µg/mL of iron element), GOx (300 µg/mL) and Fe_3_O_4_-GOx (200 µg/mL of iron element) for 5 min, respectively, in the presence of TMB and sulfuric acid. The inset shows the corresponding photographs of the four mixtures. **b** UV-vis absorption spectra of glucose solution (20 mM) with different pH values after incubation with Fe_3_O_4_-GOx (200 µg/mL of iron element) for 5 min in the presence of TMB and sulfuric acid. The inset shows the corresponding photographs of the four mixtures. **c** MB degradation in the mixture of Fe_3_O_4_-GOx (200 µg/mL of iron element) and glucose (20 mM) within 240 min in the condition of pH 5.5. The inset shows the corresponding photographs of the mixture at four scheduled time points. **d** TA fluorescent assay in the mixture of Fe_3_O_4_-GOx (200 µg/mL of iron element) and glucose (20 mM) with different pH values

The POD activity of Fe_3_O_4_-GOx and the occurrence of coupled GOx/POD cascade reaction in the system of Fe_3_O_4_-GOx/glucose were evaluated. As shown in Fig. 3a, the Fe_3_O_4_-GOx nanozyme rapidly catalyzed the oxidation of 3,3′,5,5′-tetramethylbenzidine (TMB, a POD substrate) in the presence of glucose, and produced a yellow-colored oxidation product with an absorbance maximum around 450 nm after adding sulfuric acid as the reaction termination reagent [42], showing excellent POD-like activity and confirming the occurrence of coupled GOx/POD cascade reaction. In contrast, TMB oxidation was found to be very weak in the systems of PBS/glucose, Fe_3_O_4_ NPs/glucose and GOx/glucose. Moreover, the Fe_3_O_4_-GOx/glucose system achieved a higher TMB oxidation efficiency in the conditions of pH 5.5 and pH 6.5 than that in pH 7.5 and pH 8.5, exhibiting an acidic environment-preferred GOx/POD cascade reaction (Fig. 3b). In addition to TMB oxidation, similar results were obtained using the POD substrate of 2,2′-azinobis(3-ethylbenzothiazoline-6-sulfonic acid) diammonium salt (ABTS) and o-phenylenediamine (OPD), respectively (Additional file 1: Fig. S9) [43, 44]. Theoretically, the coupled GOx/POD cascade reaction can generate the ·OH in the Fe_3_O_4_-GOx/glucose system. Therefore, the formation of ·OH in the Fe_3_O_4_-GOx/glucose system was assessed based on the methylene blue (MB) degradation and terephthalic acid (TA) fluorescent assays [45, 46]. As shown in Fig. 3c and Additional file 1: Fig. S10, blue-colored MB was gradually degraded to generate colorless MB-OH only in the system of Fe_3_O_4_-GOx/glucose, indicating the formation of ·OH. Consistent with MB degradation result, fluorescent TAOH derived from the reaction of TA and ·OH was only detected in the system of Fe_3_O_4_-GOx/glucose, and showed an acidic environment-preferred generation trend (Additional file 1: Fig. S11 and Fig. 3d). Similar results were obtained by the electron spin resonance (ESR) analysis using the spin trap molecule of DMPO to capture ·OH generated in the system of Fe_3_O_4_-GOx/glucose (Additional file 1: Fig. S12). By considering the unique pathological acidic condition of biofilm microenvironment [47, 48], the acidic pH-dependent GOx/POD cascade catalysis activity of Fe_3_O_4_-GOx to generate consecutive fluxes of toxic ·OH lays solid foundation for precise targeted biofilm elimination in DU.

### In vitro antimicrobial capability of Fe_3_O_4_-GOx

**Fig. 4:**
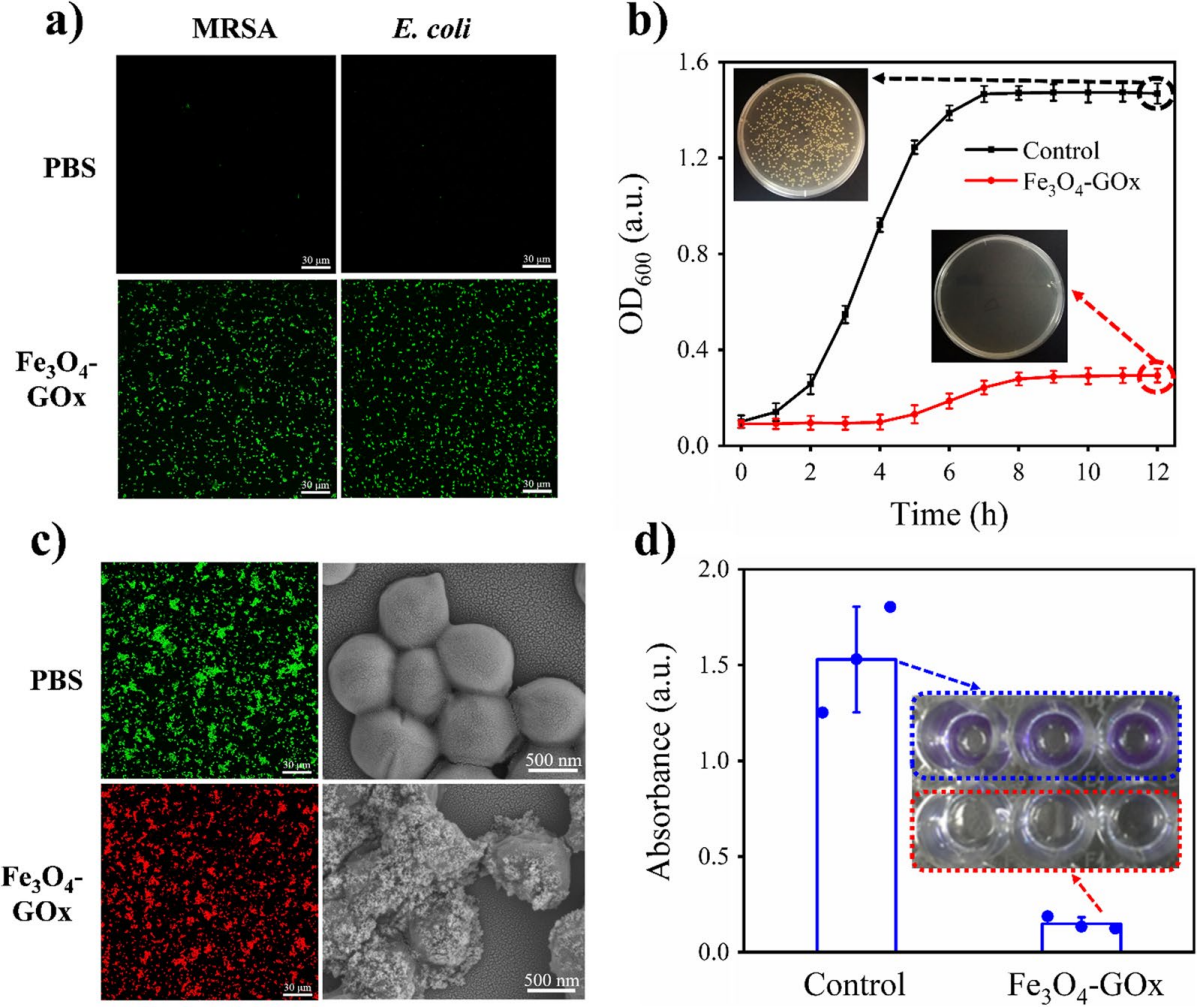
In vitro antimicrobial performance of Fe_3_O_4_-GOx. **a** Typical DCF staining images of *E. coli* and MRSA treated by the system of Fe_3_O_4_-GOx/glucose and PBS/glucose, respectively. **b** Growth curves of MRSA treated by the system of Fe_3_O_4_-GOx/glucose and control (PBS/glucose) respectively, and the inset shows the corresponding photographs of culture plates of MRSA taken from the two treatment groups at the time point of 12 h. **c** Representative SEM and live/dead staining images of MRSA treated by the system of Fe_3_O_4_-GOx/glucose and PBS/glucose, respectively. **d** Crystal violet staining image and its corresponding absorbance for integrated MRSA biofilm treated by the system of Fe_3_O_4_-GOx/glucose and control (PBS/glucose), respectively. The inset shows the corresponding photographs of crystal violet staining of MRSA biofilm in the two treatment groups. In above experiments, the concentrations of Fe_3_O_4_-GOx, glucose and bacteria used was 200 µg/mL (iron element), 20 mM and 10^7^ CFU/mL, respectively. In **b** and **d**, the values of OD_600_ and crystal violet absorbance represent the mean of three independent experiments, and the error bars indicate the SD from the mean

As an important ROS substance, ·OH produced by Fe_3_O_4_-GOx under acidic condition will greatly increase the intracellular level of ROS as well as biomacromolecules oxidative damage of bacteria, possessing robust antimicrobial capability. To verify this conclusion, dichlorodifluorescein (DCF, a fluorescent marker generated from dichlorodihydrofluorescein dye by ROS) staining as well as biomacromolecules oxidative damage assay of bacteria were carried out. Figure 4a shows the DCF staining images of bacterial strains of *Escherichia coli* (*E. coli*, gram-negative) and methicillin-resistant *Staphylococcus aureus* (MRSA, gram-positive) in the different treatment groups. It was found that *E. coli* and MRSA in the treatment group of Fe_3_O_4_-GOx/glucose exhibited much greater DCF fluorescence than the control group (PBS/glucose), indicating that the intracellular ROS level was significantly increased in bacteria after nanozyme treatment. In addition, enhanced generation of malondialdehyde (MDA) and carbonylated proteins was observed in bacteria treated by Fe_3_O_4_-GOx/glucose compared to the control (PBS/glucose), demonstrating the occurrence of more serious intracellular oxidative damage of biomacromolecules (membrane lipid and protein) after nanozyme treatment (Additional file 1: Figs. S13 and S14). The higher level of intracellular ROS as well as biomacromolecules oxidative damage will inevitably cause the death of bacteria. Therefore, the growth of *E. coli* and MRSA after nanozyme treatment was subsequently investigated, and the results are shown in Additional file 1: Fig. S15 and Fig. 4b. It was found that *E. coli* and MRSA bacterial growth was indeed extremely inhibited in the treatment group of Fe_3_O_4_-GOx/glucose compared to the control (PBS/glucose) as expected, confirming the broad-spectrum antimicrobial activity of nanozyme.

To investigate the specific mechanism behind the antimicrobial activity of Fe_3_O_4_-GOx nanozyme, live/dead bacterial staining assay and scanning electronic microscopy (SEM)-based bacterial morphology study were performed. As shown in Fig. 4c and Additional file 1: Fig. S16, *E. coli* and MRSA showed clear and smooth bodies and was stained green by the nucleic acid dye of SYTO 9 in the treatment group of PBS/glucose, exhibiting a normal survival state of living bacteria. In sharp contrast, cellular deformation and surface collapse as well as propidium iodide dye (red color, only penetrate bacteria with destroyed structure) staining were obviously found for *E. coli* and MRSA in the treatment group of Fe_3_O_4_-GOx/glucose, suggesting the bacterial cell wall and membrane disruption-involved mechanism behind the broad-spectrum antimicrobial activity of Fe_3_O_4_-GOx nanozyme [49]. This conclusion could be further proved by the experiment result of bacterial biomacromolecules leakage. As shown in Additional file 1: Figs. S17 and S18, the level of protein and DNA/RNA leakage in bacteria treated by Fe_3_O_4_-GOx/glucose was greatly improved compared to the control (PBS/glucose), indicating the structure disruption of bacteria after nanozyme treatment. Bacterial biofilm, a bacterial community wrapped by self-produced extracellular polymeric substances, is the main form of bacterial infection in DU wound [7, 50]. Therefore, in addition to planktonic bacteria, the antimicrobial activity of Fe_3_O_4_-GOx toward biofilm was also assessed. As shown in Additional file 1: Fig. S19, the formation of MRSA and *E. coli* biofilm was effectively inhibited by the Fe_3_O_4_-GOx/glucose. In addition to biofilm formation inhibition, integrated MRSA and *E. coli* biofilm were almost eradicated in the treatment group of Fe_3_O_4_-GOx/glucose while the biofilms remained intact in the control group (PBS/glucose) (Fig. 4d and Additional file 1: Fig. S20). This result demonstrates the outstanding capability of Fe_3_O_4_-GOx nanozyme for biofilm eradication and paves the way for subsequent in vivo biofilm infection treatment in DU wound.

### In vivo performance of Fe_3_O_4_-GOx for DU treatment

**Fig. 5:**
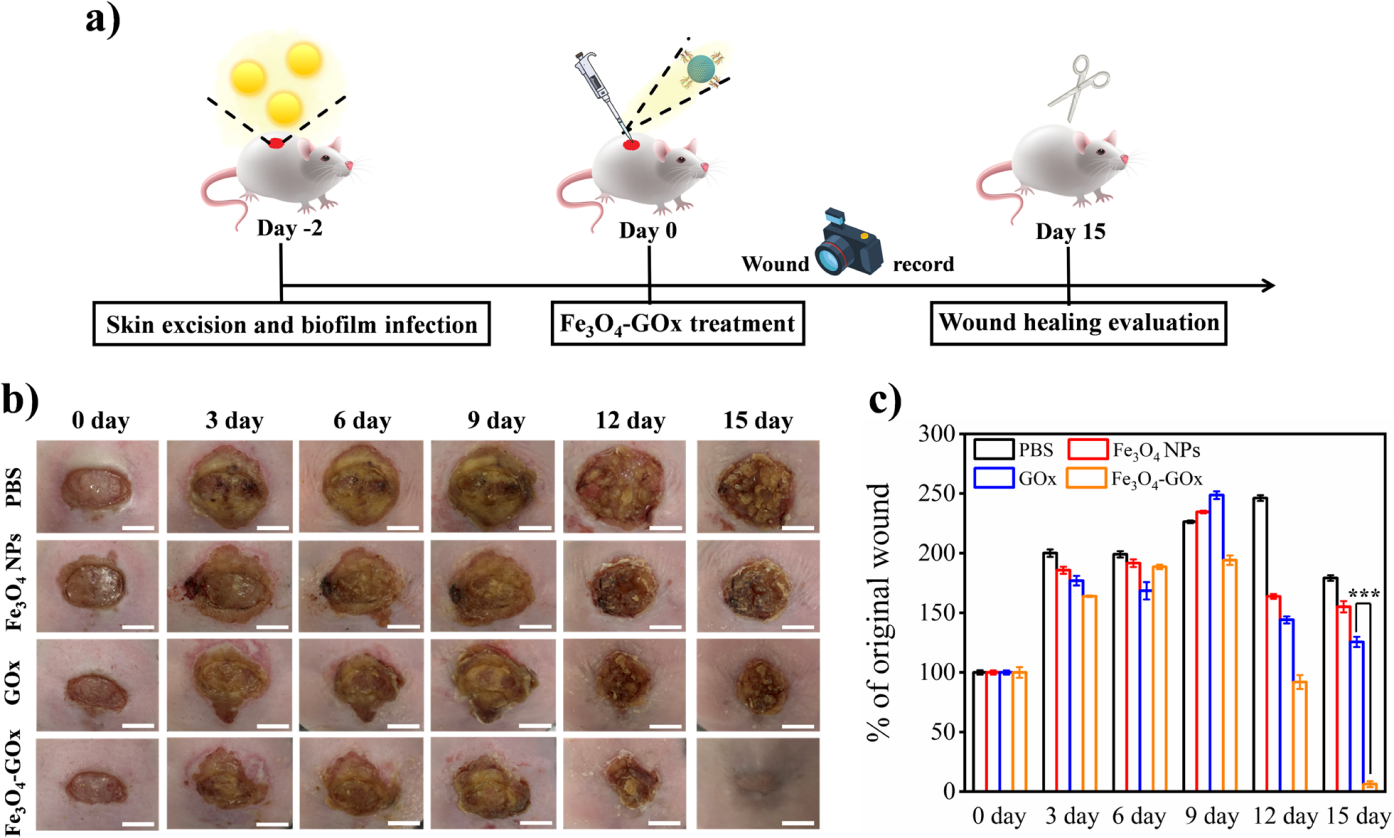
In vivo DU treatment performance of Fe_3_O_4_-GOx. **a** Experimental timeline and schematic representation of Fe_3_O_4_-GOx nanozyme for in vivo treatment of MRSA biofilm-infected diabetic wound. **b** Representative photographs of MRSA biofilm-infected diabetic wound in the treatment groups of PBS, Fe_3_O_4_ NPs, GOx, and Fe_3_O_4_-GOx within 15 days. Scale bar: 5 mm. **c** Corresponding values of wound area in the four treatment groups shown in **a**. The values of wound healing ratios (% of original wound) represent the mean of three independent experiments, and the error bars indicate the SD from the mean. ***P < 0.001

To conduct in vivo DU treatment, an MRSA biofilm-infected wound model of diabetic mouse was employed to mimic the clinical symptoms of hyperglycemia, hypoxia, excessive oxidative stress, and biofilm infection of DU. This mouse model was constructed by creating a full-thickness wound extending through the panniculus carnosus in the back of genetically modified diabetic mouse followed by MRSA inoculation to form biofilm in situ (Fig. 5a). To evaluate the performance of Fe_3_O_4_-GOx nanozyme for in vivo DU treatment, MRSA biofilm-infected diabetic wound was treated by Fe_3_O_4_-GOx and wound healing process was qualitatively and quantitatively analyzed. Four treatment groups including PBS, Fe_3_O_4_ NPs, GOx, and Fe_3_O_4_-GOx were divided in our experiments. Figure 5b shows typical photographs of MRSA biofilm-infected diabetic wound within 15 days of treatment in the four treatment groups, and the corresponding graphical representations of the quantitative measurement of wound areas are presented in Fig. 5c. It was found that compared to the treatment group of PBS, wound healing was accelerated in the other three treatment groups, and the Fe_3_O_4_-GOx nanozyme treatment group exhibited the fastest healing speed, indicating that both of the GOx and Fe_3_O_4_ NPs components of nanozyme contribute to the healing of infected diabetic wound. This result is consistent with our hypothesis that Fe_3_O_4_-GOx nanozyme with closely co-localized GOx and Fe_3_O_4_ NPs can synergistically address the issues of hyperglycemia, hypoxia, oxidative stress and biofilm infection, reshaping the pathological wound microenvironment and rescuing the stalled healing in DU. Compared with photodynamic antimicrobial chemotherapy commonly used in chronic wound treatment [51], Fe_3_O_4_-GOx nanozyme exhibits comparable broad-spectrum antimicrobial performance as well as wound healing rate without the help of light irradiation, addressing the shortcomings of conventional phototherapy (e.g., limited light penetration depth, and wound hypoxia). Furthermore, apart from outstanding anti-oxidant activity similar to the commonly used anti-oxidant hydrogel [52], the Fe_3_O_4_-GOx also possessed additional pH-switchable antimicrobial capability, which makes it more suitable for DU wound treatment.

**Fig. 6:**
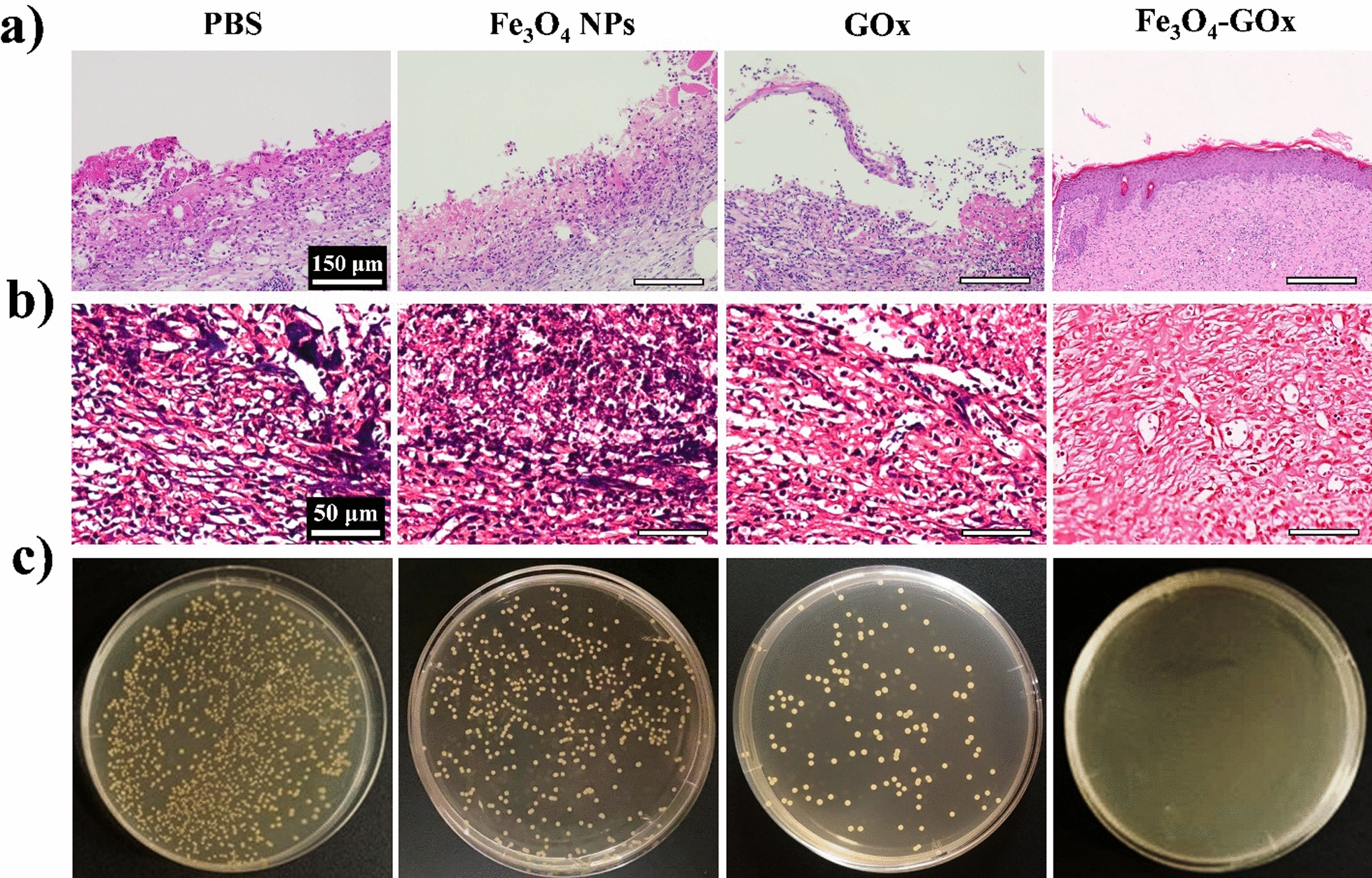
Representative hematoxylin and eosin (HE) staining (**a**), gram-staining (**b**), and bacterial culture plate (**c**) images of MRSA biofilm-infected diabetic wound after 15 days treatment of PBS, Fe_3_O_4_ NPs, GOx, and Fe_3_O_4_-GOx, respectively

**Fig. 7:**
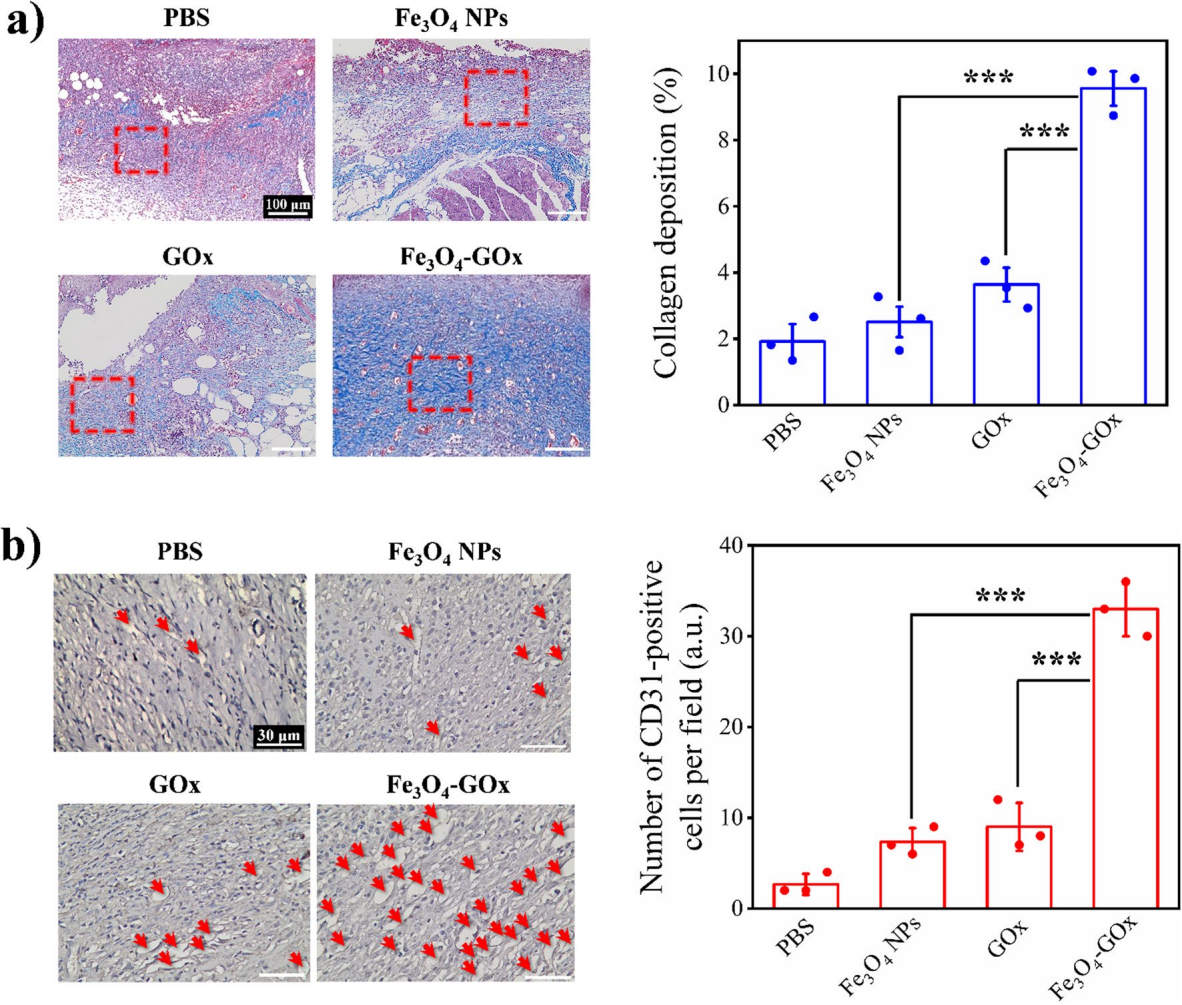
Collagen deposition and blood vessel formation in DU wound under different treatment. **a** Representative Masson’s trichrome staining images and the corresponding quantitative data of collagen deposition percentage of the tissues of MRSA biofilm-infected diabetic wound in four treatment groups. The red rectangle parts in the images are magnified in Additional file 1: Fig. S17 to show the condition of fiber alignment. **b** Representative CD31 staining images and the corresponding quantitative data of the CD34-positive cell’s number of the tissues of MRSA biofilm-infected diabetic wound in four treatment groups indicate the condition of blood vessel formation. In **a** and **b**, the values of collagen deposition percentage and CD31-positive cell’s number determined using 9 staining images from three independent experiments are stated as mean ± SD. ***P < 0.001

Subsequently, histological analysis of wound tissues in the four treatment groups was carried out. Similar to normal skin tissue (Additional file 1: Fig. S21), morphological features of blood vessels and hair follicles was observed in the infected diabetic wound tissue after 15 days of Fe_3_O_4_-GOx treatment, indicating the complete re-epithelialization of wound (Fig. 6a). In addition, gram-staining and bacterial culture of the wound tissues in the four treatment groups were performed to evaluate the performance of Fe_3_O_4_-GOx for in vivo biofilm eradication. As shown in Fig. 6b, c, bacterial load in wound tissues was positively correlated with the healing of wound, and complete biofilm elimination was only obtained in the treatment group of Fe_3_O_4_-GOx on the 15th day of treatment. This phenomenon means that the inflammation phase of wound healing in DU will be greatly shortened by the treatment of Fe_3_O_4_-GOx nanozyme to recover the normal orchestrated course of wound-healing cascades [53]. Furthermore, the collagen deposition as well as blood vessel density of wound tissues in the four treatment groups were investigated. It was found that Fe_3_O_4_-GOx treatment accelerated the collagen deposition (light blue color in Fig. 7a), fiber alignment (Additional file 1: Fig. S22), and blood vessel formation (red arrows in Fig. 7b) of wound tissues compared to the other three treatment groups based on Masson’s trichrome and immunohistochemistry CD31 staining assays. The collagen deposition, fiber alignment and blood vessels formation of wound tissues on the 15th day of Fe_3_O_4_-GOx treatment was almost same to that of normal skin tissues (Additional file 1: Fig. S23), demonstrating that the Fe_3_O_4_-GOx nanozyme also has a notable accelerated effect on the proliferation and remodeling courses of wound healing besides shortening the inflammation phase [54]. These multi-dimensional experimental results describe above strongly prove the feasibility of Fe_3_O_4_-GOx nanozyme for in vivo DU treatment to rescue the stalled wound healing. In addition to the mice model of biofilm-infected diabetic wound, the in vivo performance of Fe_3_O_4_-GOx nanozyme for the healing of non-infected diabetic wound was further evaluated. As shown in Additional file 1: Fig. S24, compared with the control group (PBS), the Fe_3_O_4_-GOx treated non-infected diabetic wounds healed completely on the 10th day of treatment. Moreover, the Fe_3_O_4_-GOx was found to accelerate the collagen deposition, blood vessel formation, and tissue re-epithelization of non-infected diabetic wound based on the results of Masson’s trichrome staining (Additional file 1: Fig. S25), CD 31 staining (Additional file 1: Fig. S26) and HE analysis (Additional file 1: Fig. S27). These results demonstrate that Fe_3_O_4_-GOx can greatly accelerate the healing of non-infected diabetic wound via the GOx/CAT cascade reaction, which means that the gluconic acid produced in the oxidation process of glucose has negligible effect on the in vivo performance of Fe_3_O_4_-GOx nanozyme.

The potential biotoxicity of nanomaterials is a key obstacle to its clinical transformation [55]. By considering the outstanding biocompatibility of the components of Fe_3_O_4_ NPs (an FDA-approved nanomaterial) and GOx (a natural protein), the Fe_3_O_4_-GOx nanozyme has predictable excellent biosafety in vitro and in vivo. By performing antiproliferation assay, HUVEC cells were found to possess high viability after incubation with Fe_3_O_4_-GOx nanozyme, Fe_3_O_4_ NPs, and GOx respectively for different times, confirming their wonderful biocompatibility in vitro (Additional file 1: Fig. S28). To evaluate the biosafety effect of Fe_3_O_4_-GOx in vivo, healthy diabetic mice were subcutaneously injected with Fe_3_O_4_-GOx nanozyme, Fe_3_O_4_ NPs, GOx, and PBS (control), respectively, and the blood biochemical assay as well as organ histopathological analysis was carried out on the 7th day of injection. As summarized in Additional file 1: Fig. S29, no obvious difference of blood biochemical indicators detected was found in the four groups of injection, indicating the negligible damage of Fe_3_O_4_-GOx to the metabolism of liver and kidney of mice. Moreover, no lesions and inflammation were found in the main organs of the mice treated by Fe_3_O_4_-GOx compared to the control from the histopathological staining images, exhibiting outstanding in vivo biocompatibility (Additional file 1: Fig. S30). This confirmed excellent in vitro and in vivo biosafety of Fe_3_O_4_-GOx nanozyme lay a solid foundation for its future clinical transformation.

## Conclusions

In summary, we report a PNCC strategy for spatial–temporal modulation of pathological wound microenvironment to rescue stalled healing in DU by employing Fe_3_O_4_-GOx nanozyme. The Fe_3_O_4_-GOx possesses GOx, CAT and POD activities, and can program pH-switchable GOx/POD and GOx/CAT cascade reaction in neutral and acidic condition, respectively. The GOx/POD cascade reaction generating consecutive fluxes of toxic hydroxyl radical spatially targets the acidic biofilm (pH ~ 5.5), and eradicates biofilm to shorten the inflammatory phase and initiate the normal healing course. Furthermore, the GOx/CAT cascade reaction producing consecutive fluxes of oxygen spatially targets the neutral wound tissue, and accelerates the proliferation and remodeling phases of wound healing by addressing the issues of hyperglycemia, hypoxia, and excessive oxidative stress. Notably, the shortened inflammatory phase temporally coupled with accelerated proliferation and remodeling phases significantly speed up the normal orchestrated wound-healing cascades, enables complete in vivo re-epithelialization of biofilm-infected diabetic wound within 15 days, demonstrating strong capability for in vivo DU management. More importantly, inheriting the excellent biocompatibility from the GOx and clinically approved Fe_3_O_4_ NPs, the Fe_3_O_4_-GOx nanozyme exerts great potential for clinical transformation. We believe that the proposed PNCC concept offers a new perspective for complex pathological microenvironment remodeling, and may provide a powerful modality for the treatment of pathological microenvironment-associated diseases.

## Materials and methods

### Chemicals and materials

Iron (III) chloride hexahydrate (FeCl_3_·6H_2_O), sodium oleate, oleic acid, 1-octadecene, poly (allylamine hydrochloride) (PAH; *M*_w_ = 15,000 Da), phosphate buffered saline (PBS), tetramethylammonium hydroxide, *N*-(3-(dimethylamino)propyl-*N*′-ethylcarbodiimide) hydrochloride (EDC), *N*-hydroxysulfosuccinimide sodium salt (NHS), 3-(4,5-dimethyl-2-thiazolyl)-2,5-diphenyl-2 H-tetrazolium bromide (MTT) assay kit and 2ʹ,7ʹ-dichlorohydrofluorescein diacetate (DCFH; ≥ 94%), glucose oxidase (GOx) were purchased from Sigma-Aldrich. 3,3′,5,5′-tetramethylbenzidine (TMB), 2,2′-azinobis (3-ethylbenzothiazoline-6-sulfonic acid) diammonium salt (ABTS), o-phenylenediamine (OPD; 98%), methylene blue (MB), terephthalic acid (TA) and hydrogen peroxide (H_2_O_2_) assay kit were obtained from Beyotime Biotechnology. Bicinchoninic acid (BCA) protein assay kit, malondialdehyde (MDA) and protein carbonyl assay kit were obtained from Nanjing Jiancheng Institute of Biological Engineering. Live/dead bacteria viability kit was purchased from Thermo Fisher Scientific. All other chemicals were obtained from Adamas-beta and used without further purification. Deionized (DI) water (Millipore Milli-Q grade, 18.2 MΩ) was used in all the experiments.

### Preparation of Fe_3_O_4_-GOx nanozyme

Magnetic Fe_3_O_4_ NPs were first synthesized by thermal decomposition of the iron–oleate complex according to previously reported methods [38, 39]. Briefly, 12.2 g of sodium oleate and 3.6 g of FeCl_3_·6H_2_O were dissolved in the mixed solution of DI water, hexane and absolute ethanol, and refluxed at 70 °C for 4 h. Then, the upper organic liquid obtained by the liquid separation was washed with DI water, and hexane was removed to obtain a brown-red iron oleate complex. Next, 6 g of iron oleate complex was dissolved in octadecene and oleic acid mixed solution under the protection of N_2_. The mixture was then heated to 320 °C, refluxed and condensed for 30 min to obtain magnetic Fe_3_O_4_ NPs. In order to make the prepared Fe_3_O_4_ NPs water-soluble, absolute ethanol was added to Fe_3_O_4_ NPs dispersed in hexane followed by magnet separation, and tetramethylammonium hydroxide and propanol were subsequently added. After 5 min shaking, the mixture was separated by magnet, and water-soluble Fe_3_O_4_ NPs was obtained by acetone wash and finally dispersed in DI water.

To prepare Fe_3_O_4_-GOx nanozyme, cationic PAH polyelectrolyte was first used to coat the negatively charged Fe_3_O_4_ NPs, and the carboxyl group of GOx molecules was sequentially conjugated with the amino group of PAH through a chemical covalent coupling method [56, 57]. In brief, 0.1 g of PAH was dissolved in 10 mL of NaCl solution (1 mM), and 1 mL of as-prepared Fe_3_O_4_ NPs were added. The mixture was stirred at 900 rpm for 3 h at room temperature, and the PAH-coated Fe_3_O_4_ NPs (Fe_3_O_4_/PAH) were collected by centrifugation at 12,000 rpm for 15 min and dispersed in 1 mL of DI water for GOx modification. First, 4.1 mg EDC and 4.5 mg NHS were added to 1 mL of 10 mg/mL GOx to activate the carboxyl group. After 30 min, 1 mL of Fe_3_O_4_/PAH were added to the mixture, and stirred at 900 rpm for 24 h at room temperature. After the reaction was completed, the mixture was centrifuged at 12,000 rpm for 15 min, and the precipitate (Fe_3_O_4_-GOx nanozyme) was washed with DI water, and finally resuspended in DI water for further characterization and application.

### In vitro biocompatibility assay

The biocompatibility of Fe_3_O_4_-GOx nanozyme was determined by MTT assay using human umbilical vein endothelial cells (HUVEC). In brief, HUVEC cells were seeded into a 96-well plate (8000–10,000 cells/well) and cultured overnight. Then the cells were treated with Fe_3_O_4_-GOx nanozyme with different concentrations (0, 5, 10, 50, 100, and 200 µg/mL of iron element). After 24 h of culture, MTT reagent was added and the cell viability was evaluated with a microplate reader.

### pH-switchable cascade catalysis performance of Fe_3_O_4_-GOx nanozyme

The GOx activity (oxidize glucose to produce H_2_O_2_) of Fe_3_O_4_-GOx nanozyme was evaluated by glucose depletion and H_2_O_2_ generation. For glucose depletion assay, Fe_3_O_4_-GOx nanozymes with different concentrations (0, 5, 10, 20, 50, 100, and 200 µg/mL of iron element) were incubated with diabetic blood sample containing around 20 mM glucose for 5 min, and the blood glucose concentration was measured with a glucometer. For H_2_O_2_ generation assay, Fe_3_O_4_-GOx nanozymes with different concentrations (0, 5, 10, 20, 50, 100, and 200 µg/mL of iron element) were incubated with 20 mM glucose solution for different times (5, 10, 30, 60, 90, 120 and 240 min). Then the mixtures were incubated with H_2_O_2_ detecting reagent for 30 min, and the amount of H_2_O_2_ generated was determined by measuring the absorbance of the mixture at a wavelength of 560 nm.

The CAT activity of Fe_3_O_4_-GOx nanozyme and the occurrence of coupled GOx/CAT cascade reaction was evaluated by demonstrating the generation of oxygen in the system of Fe_3_O_4_-GOx/glucose. Briefly, Fe_3_O_4_-GOx nanozymes (200 µg/mL of iron element) was co-cultured with glucose solution (20 mM) for 5 min under different pH conditions (5.5, 6.5, 7.5 and 8.5), and oxygen probes (JPBJ-608 portable Dissolved Oxygen Meters, Shanghai REX Instrument Factory) was used to detect the amount of oxygen generated in the mixture.

The POD activity of Fe_3_O_4_-GOx nanozyme and the occurrence of coupled GOx/POD cascade reaction was assessed by the oxidation of POD substrates of TMB, ABTS and OPD respectively, in the system of Fe_3_O_4_-GOx/glucose. Briefly, Fe_3_O_4_-GOx nanozymes (200 µg/mL of iron element) was co-cultured with glucose solution (20 mM) for 5 min, and POD substrate (TMB, ABTS or OPD) was then added and incubated for 30 min under different pH conditions (5.5, 6.5, 7.5 and 8.5). The oxidation of TMB (using sulfuric acid as a stop reagent), ABTS, and OPD was evaluated by measuring the absorbance of the mixture at the wavelength of 450, 734, and 492 nm, respectively.

### Measurement of hydroxyl radical

MB degradation and TA fluorescent assay were employed to assess the ability of Fe_3_O_4_-GOx nanozyme to generate ·OH after incubation with glucose. MB can be degraded by ·OH to generate MB-OH, while TA can react with ·OH to generate fluorescent TAOH. In typical experiments, Fe_3_O_4_-GOx nanozymes (200 µg/mL of iron element) was co-cultured with glucose solution (20 mM) for 5 min, then MB degradation and TA fluorescent assay were performed respectively. In MB degradation, the mixture of Fe_3_O_4_-GOx nanozyme and glucose was incubated with MB (10 µg/mL) for 24 h, and the absorbance of the mixture at the wavelength of 664 nm was recorded. In TA fluorescent assay, the mixture of Fe_3_O_4_-GOx nanozyme and glucose was incubated with TA (0.5 mM) for 12 h, and the fluorescence spectrum of the mixture was measured.

### Bacteria culture and antimicrobial experiments

Methicillin-resistant *Staphylococcus aureus* (MRSA) (ATCC 33591) and *Escherichia coli* (*E. coli*) (ATCC 8739) were used in our experiments. MRSA and *E. coli* were cultured in tryptic soy broth (TSB) medium and lysogeny broth (LB) medium, respectively, and harvested at the exponential growth phase before use. For antimicrobial experiments, 10^6^ CFU of bacteria were incubated with the mixture of Fe_3_O_4_-GOx nanozyme (200 µg/mL of iron element) and glucose (20 mM) for 2 h in the condition of pH 6.5, and the antimicrobial performance was evaluated by the live/dead bacterial staining assay and SEM-based bacterial morphology investigation.

#### Live/dead bacterial staining assay

Live/dead staining assay kit was used to evaluate the viability of bacteria. In brief, the bacteria before and after Fe_3_O_4_-GOx nanozyme treatment were mixed with the dye solution containing SYTO 9 and propidium iodide for 30 min in the dark, and then imaged with a confocal fluorescence microscope. Live bacteria were stained by SYTO 9 with green color, while dead bacteria were stained by propidium iodide with red color due to the damage of cell membrane and wall [58].

### SEM-based morphological study of bacteria

The morphology of bacteria was characterized by field-emission scanning electron microscopy (FESEM). In brief, the bacteria before and after Fe_3_O_4_-GOx nanozyme treatment were fixed with glutaraldehyde (2.5%) in the dark for 2 h, and dehydrated by ethanol solution with different concentrations (50%, 70%, 90% and 100%) for 10 min. The dehydrated bacterial samples were dropped on silicon wafer, and imaged with FESEM after nitrogen drying and platinum coating.

### Mice model of DU

Type II diabetic mice (db/db, 6 weeks, ∼ 40 g) were purchased from the Nanjing Si Ke Rui Biological Technology Co., Ltd., and allowed to acclimatize for 1 week in the laboratory. All animal experiments were carried out in compliance with the protocols approved by the Shandong University Laboratory Animal Center. To construct the mouse model of DU, the diabetic mouse was firs anesthetized, and an oval wound (12 mm in long axis, 9 mm in short axis) was created on the back of mouse using disposable biopsy punch followed by bacteria (10^7^ CFU of *E. coli* or MRSA) inoculation for 2 days before treatment to form biofilm in situ.

### In vivo DU treatment

The MRSA biofilm-infected wound of diabetic mice were divided into four treatment groups including Fe_3_O_4_-GOx, Fe_3_O_4_ NPs, GOx and PBS, and each treatment group contained 5 mice. To carry out in vivo DU treatment, materials (Fe_3_O_4_-GOx, Fe_3_O_4_ NPs, GOx or PBS) were added onto the infected diabetic wound, and pictures of the wound were taken every day to record their festered areas. After 15 days treatment, the mice were executed and the skin tissues at the wound sites were dissected. A variety of experimental methods including bacteria culture, hematoxylin and eosin (HE) staining, Masson’s trichrome staining, gram staining and CD31 immunohistochemistry staining, were performed to evaluate the actual therapeutic ability of Fe_3_O_4_-GOx nanozyme for in vivo DU treatment.

## Supplementary Information

**Additional file 1.** Additional experimental methods and figures.

## Data Availability

All data generated or analyzed during this study are included in this published article.
