# Supplementary material for: pH-switchable nanozyme cascade catalysis: a strategy for spatial–temporal modulation of pathological wound microenvironment to rescue stalled healing in diabetic ulcer
Source: J Nanobiotechnology. 2022 Jan 4;20:12. doi: 10.1186/s12951-021-01215-6 (PMC8725300; doi:10.1186/s12951-021-01215-6)
Supplement: Supplementary file 1 — Additional file 1. Additional experimental methods and figures. [file 12951_2021_1215_MOESM1_ESM.docx]

**Additional Information**

**pH-switchable nanozyme cascade catalysis: a strategy for spatial-temporal modulation of pathological wound microenvironment to rescue stalled healing in diabetic ulcer**

Xuancheng Du^1†^, Bingqing Jia^1†^, Weijie Wang^1^, Chengmei Zhang^2^, Xiangdong Liu^1^, Yuanyuan Qu^1^, Mingwen Zhao^1^, Weifeng Li^1^, Yanmei Yang^3*^, Yong-Qiang Li^1,4*^

^1^ Institute of Advanced Interdisciplinary Science, School of Physics, Shandong University, Jinan 250100, China.

^2^ Laboratory Animal Center of Shandong University, Jinan 250012, China.

^3^ College of Chemistry, Chemical Engineering and Materials Science, Collaborative Innovation Center of Functionalized Probes for Chemical Imaging in Universities of Shandong, Key Laboratory of Molecular and Nano Probes, Ministry of Education, Shandong Normal University, Jinan, 250014, China.

^4^ Suzhou Research Institute, Shandong University, Suzhou 215123, China.

Experimental

Determination of ROS in bacteria

2ʹ,7ʹ-Dichlorofluorescin Diacetate (DCFH-DA) was used to determine the level of ROS in bacteria. In brief, bacteria before and after Fe_3_O_4_-GOx nanozyme treatment were first incubated with DCFH-DA (10 μM) for 30 min in dark, respectively, and washed three times with PBS after centrifugation. Then bacteria were imaged by a confocal fluorescence microscope, and their fluorescence spectrums were recorded using a microplate reader.

Assessment of membrane lipid oxidation and carbonylated protein generation in bacteria

To verify the destruction of important biomolecules in bacterial cells after Fe_3_O_4_-GOx nanozyme treatment, the oxidation of membrane lipid and the generation of carbonylated protein were assessed. Briefly, bacteria samples before and after Fe_3_O_4_-GOx nanozyme treatment were processed with the malondialdehyde (MDA) and protein carbonyl assay kits respectively. The absorbance of bacterial samples at the wavelength of 532 nm in MDA assay was recorded to assess the degree of membrane lipid oxidation, while the absorbance of bacterial samples at the wavelength of 370 nm in protein carbonyl assay was recorded to assess the degree of carbonylated protein generation.

Evaluation of the leakage of intracellular components in bacteria

The leakage of intracellular components (protein and DNA/RNA) in bacteria after Fe_3_O_4_-GOx nanozyme treatment was evaluated. Bacterial samples before and after Fe_3_O_4_-GOx nanozyme treatment were processed with the BCA protein assay kit, respectively, and their absorbance at 562 nm was measured to assess the degree of intracellular protein leakage in bacteria. In addition, the absorbance of bacteria samples before and after Fe_3_O_4_-GOx nanozyme treatment at the wavelength of 260 nm were recorded, respectively, to demonstrate the degree of intracellular DNA/RNA leakage in bacteria.

*In vivo* biosafety investigation of Fe_3_O_4_-GOx nanozyme

Organ pathological examination and blood biochemistry assay were conducted to evaluate the *in vivo* biosafety of Fe_3_O_4_-GOx nanozyme. In brief, Fe_3_O_4_-GOx (200 μg/mL) was subcutaneously injected into type II diabetic mice, and major organs and blood samples were collected on the 7^th^ day of injection. HE staining of organs and blood biochemistry assay were performed to demonstrate the *in vivo* biocompatibility of Fe_3_O_4_-GOx nanozyme. Organ pathological examination and blood biochemistry assay of diabetic mice injected with PBS were used as the control.

Statistical analysis

Data are expressed as mean ± standard deviation. Student’s two-tailed *t* tests was performed for statistical analysis, * indicates *p* < 0.05, ** indicates *p* < 0.01, and *** indicates *p* < 0.001.

Additional figures


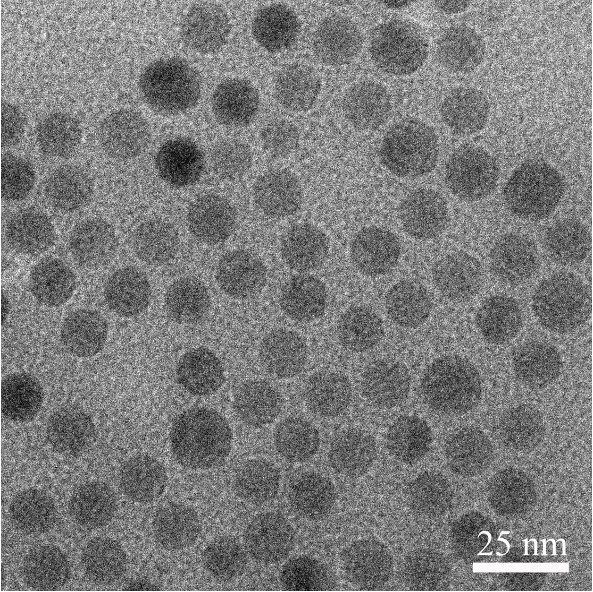


**Fig. S1**  TEM image of synthesized Fe_3_O_4_ NPs.


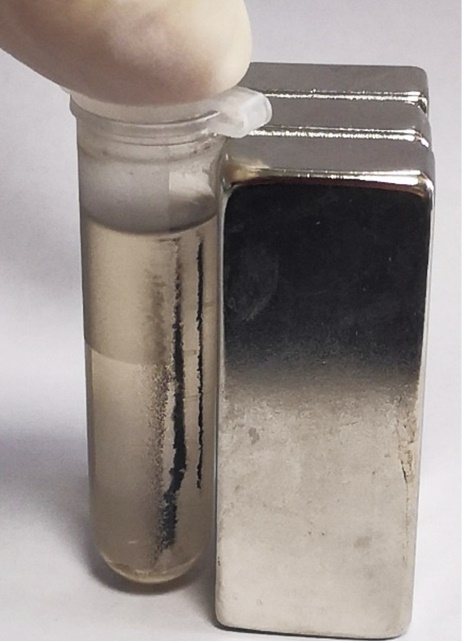


**Fig. S2** Photograph of Fe_3_O_4_ NPs solution with an external magnet imposition.


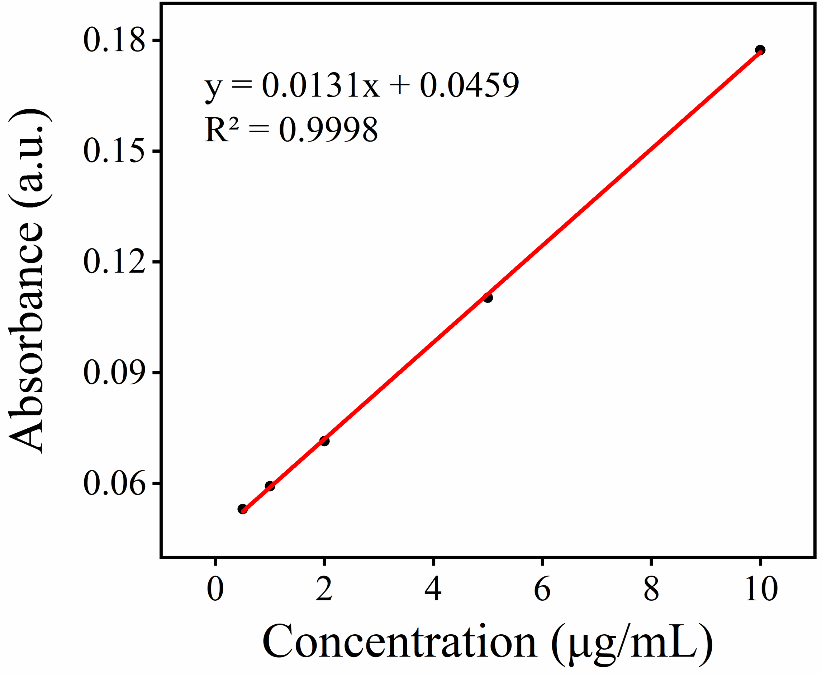


**Fig. S3** The linear calibration curve between GOx concentration and its absorbance in BCA assay.


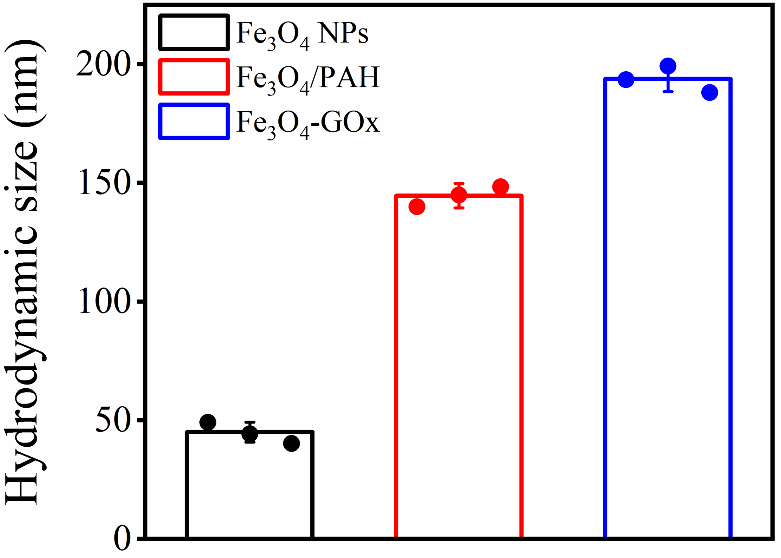


**Fig. S4** Hydrodynamic diameter of Fe_3_O_4_ NPs, Fe_3_O_4_/PAH, and Fe_3_O_4_-GOx nanozyme in PBS buffer (0.01 M, pH 7.4). The values of hydrodynamic diameter represent the mean of three independent experiments, and the error bars indicate the standard deviation (SD) from the mean.





**Fig. S5** Hydrodynamic diameter of Fe_3_O_4_-GOx nanozyme in PBS buffer (0.01 M, pH 7.4) during two weeks of storage at 4 and 25 °C, respectively. The values of hydrodynamic size represent the mean of three independent experiments, and the error bars indicate SD from the mean.


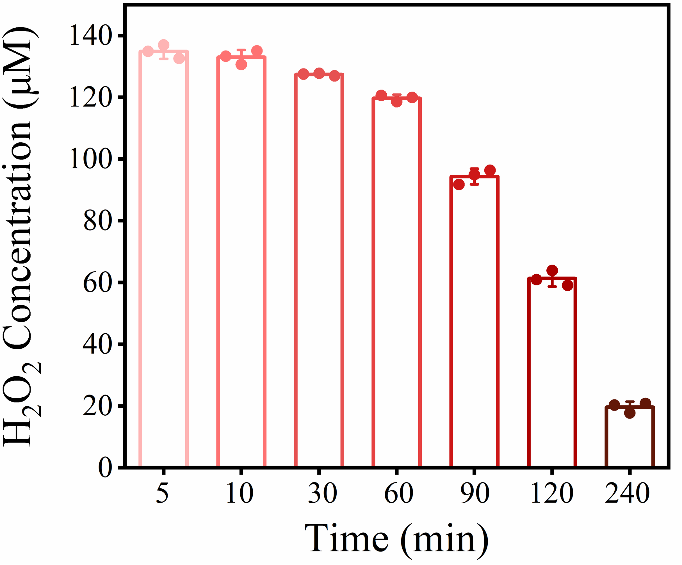


**Fig. S6** The concentrations of H_2_O_2_ generated in glucose solution (20 mM, pH 7.5) after incubation with Fe_3_O_4_-GOx nanozyme (200 µg/mL of iron element) for different times. The value of H_2_O_2_ concentrations represent the mean of three independent experiments, and the error bars indicate the SD from the mean.





**Fig. S7** XPS spectrum of Fe_3_O_4_-GOx.





**Fig. S8** The pH changes of PBS/glucose, Fe_3_O_4_/glucose and Fe_3_O_4_-GOx/glucose systems within 60 min. The working concentration of glucose was 20 mM.


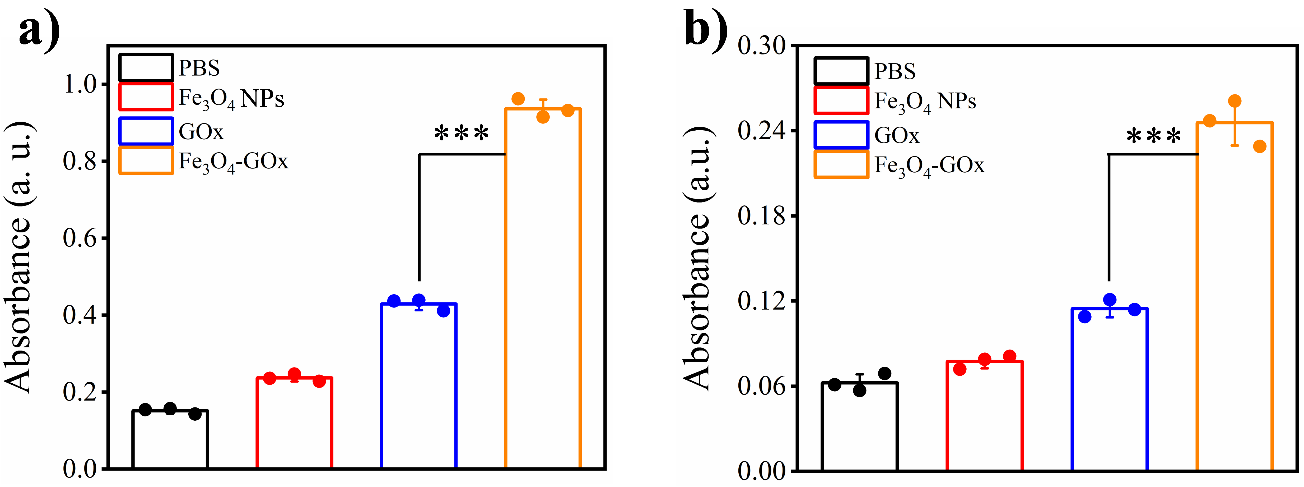


**Fig. S9**  UV-vis absorption spectra of glucose solution (20 mM, pH 5.5) after incubation with PBS, Fe_3_O_4_ NPs (200 µg/mL of iron element), GOx (300 µg/mL) and Fe_3_O_4_-GOx (200 µg/mL of iron element) for 5 min, respectively, in the presence of ABTS (a) and OPD (b). The values of absorbance represent the mean of three independent experiments, and the error bars indicate the SD from the mean. ****P* < 0.001.


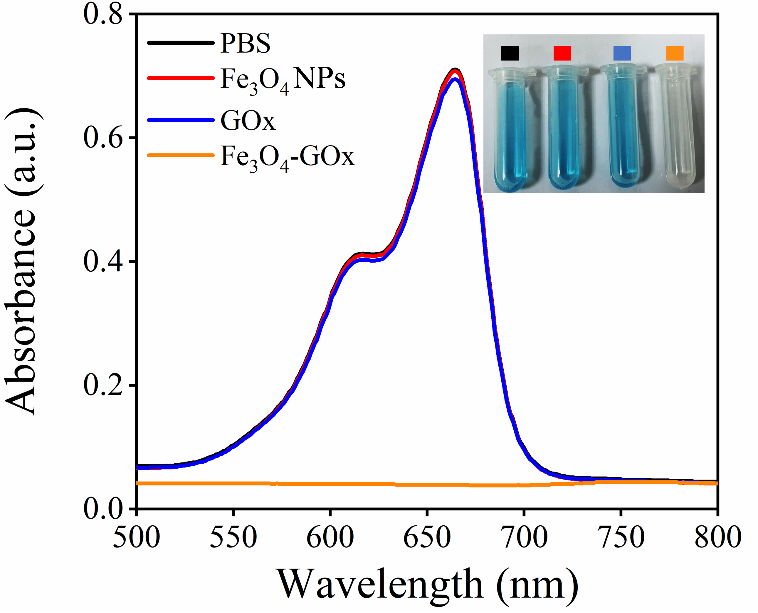


**Fig. S10**  MB degradation in the glucose solution (20 mM, pH 5.5) incubated with PBS, Fe_3_O_4_ NPs (200 µg/mL of iron element), GOx (300 µg/mL) and Fe_3_O_4_-GOx (200 µg/mL of iron element), respectively. The inset shows the corresponding photographs of the four mixtures.


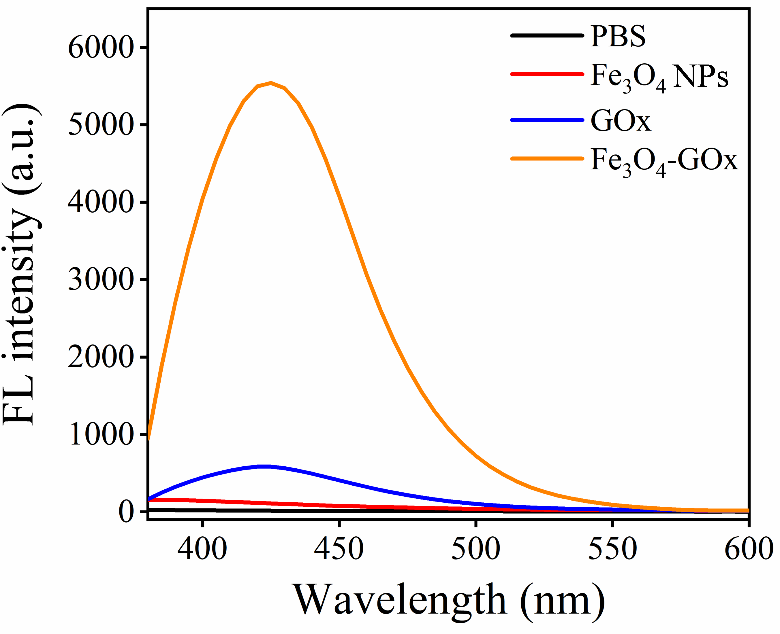


**Fig. S11** TA fluorescent assay in the glucose solution (20 mM, pH 5.5) incubated with PBS, Fe_3_O_4_ NPs (200 µg/mL of iron element), GOx (300 µg/mL) and Fe_3_O_4_-GOx (200 µg/mL of iron element), respectively.





**Fig. S12** ESR spectra of the mixture of Fe_3_O_4_-GOx and glucose with different pH values by using the spin trap molecule of DMPO to capture generated ·OH.


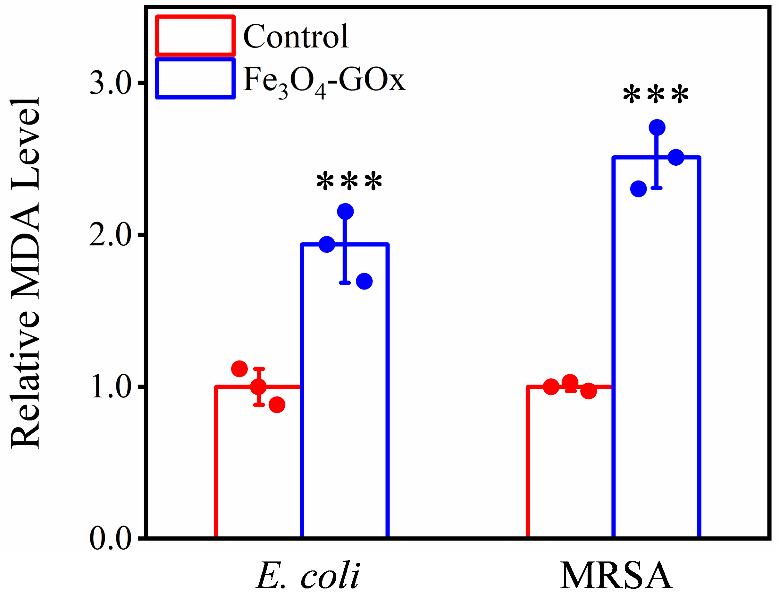


**Fig. S13**  The generation level of malondialdehyde (MDA) in *E. coli* and MRSA in the treatment group of Fe_3_O_4_-GOx/glucose and control (PBS/glucose), respectively. The values of MDA level represent the mean of three independent experiments, and the error bars indicate the SD from the mean. ****P* < 0.001.


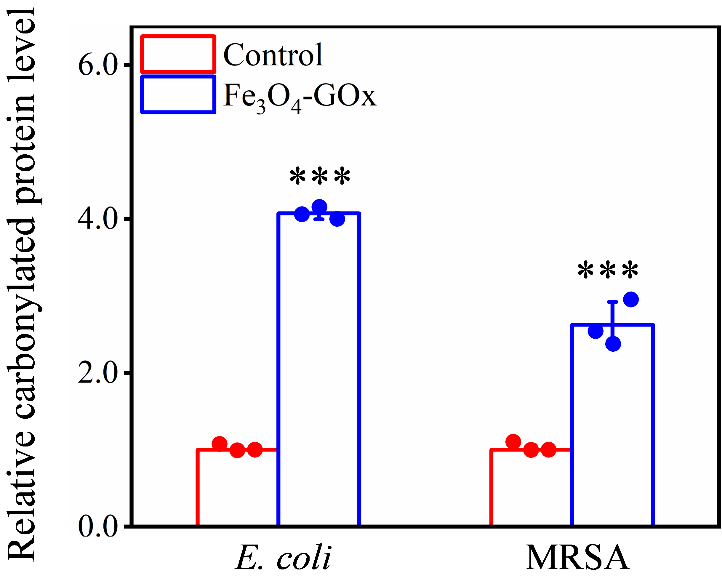


**Fig. S14** The generation level of carbonylated protein in *E. coli* and MRSA in the treatment group of Fe_3_O_4_-GOx/glucose and control (PBS/glucose), respectively. The values of carbonylated protein level represent the mean of three independent experiments, and the error bars indicate the SD from the mean. ****P* < 0.001.


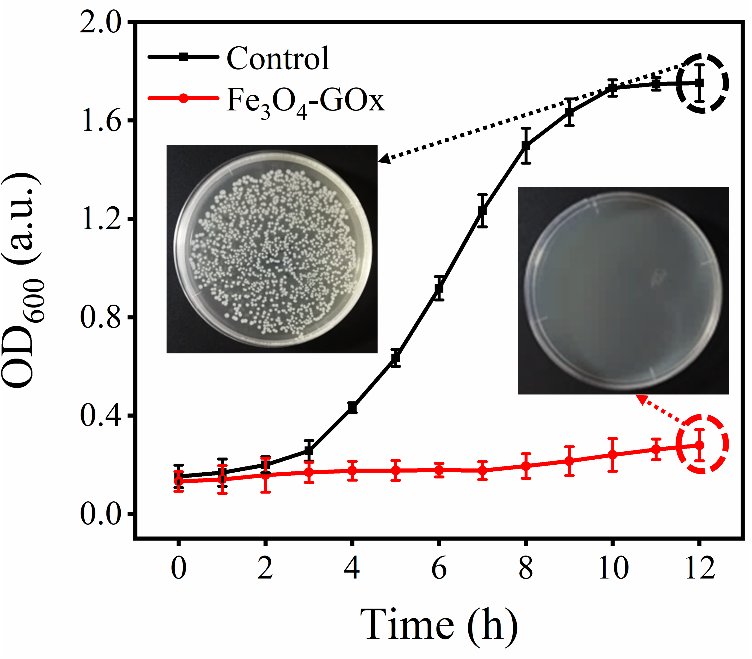


**Fig. S15**  Growth curves of *E. coli* treated by the system of Fe_3_O_4_-GOx/glucose and control (PBS/glucose) respectively, and the inset shows the corresponding photographs of culture plates of *E. coli* taken from the two treatment groups at the time point of 12 h. The values of OD_600_ represent the mean of three independent experiments, and the error bars indicate the SD from the mean.


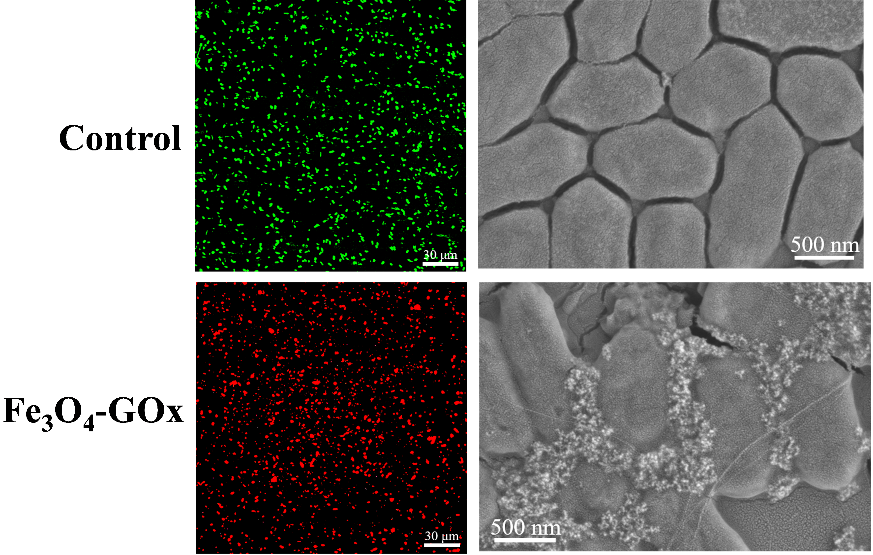


**Fig. S16** Representative SEM and live/dead staining images of *E. coli* treated by the system of Fe_3_O_4_-GOx/glucose and PBS/glucose, respectively.


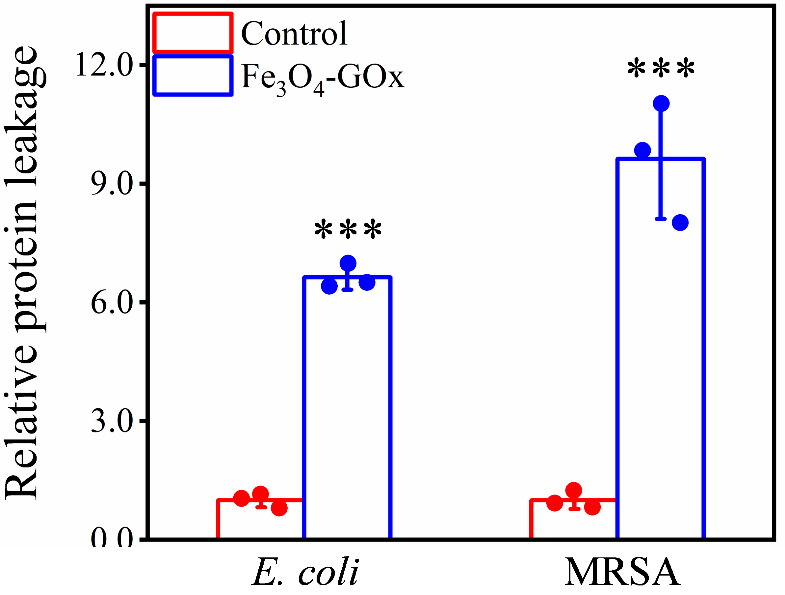


**Fig. S17** The level of protein leakage in bacteria treated by the system of Fe_3_O_4_-GOx/glucose and control (PBS/glucose), respectively. The values of relative protein leakage represent the mean of three independent experiments, and the error bars indicate the SD from the mean. ****P* < 0.001.


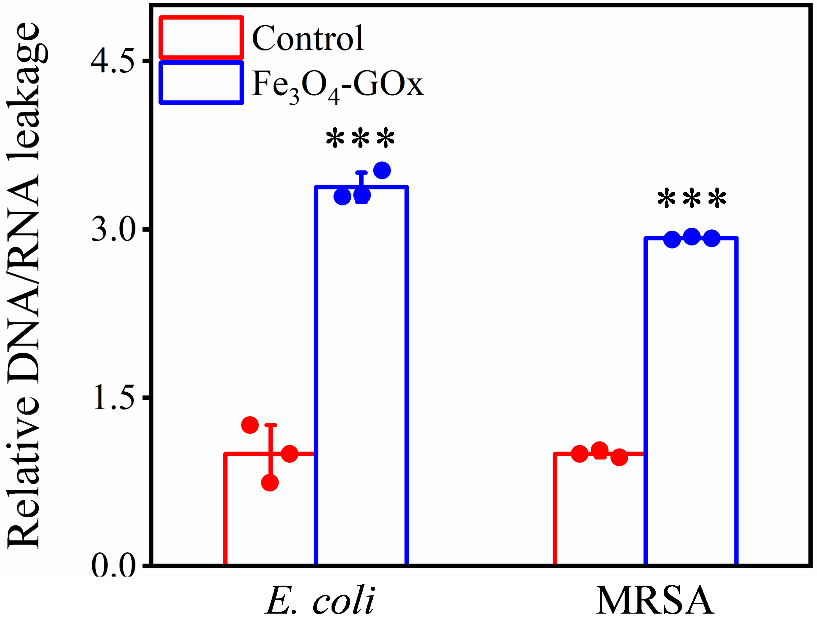


**Fig. S18** The level of DNA/RNA leakage in bacteria treated by the system of Fe_3_O_4_-GOx/glucose and control (PBS/glucose), respectively. The values of relative DNA/RNA leakage represent the mean of three independent experiments, and the error bars indicate the SD from the mean. ****P* < 0.001.

**
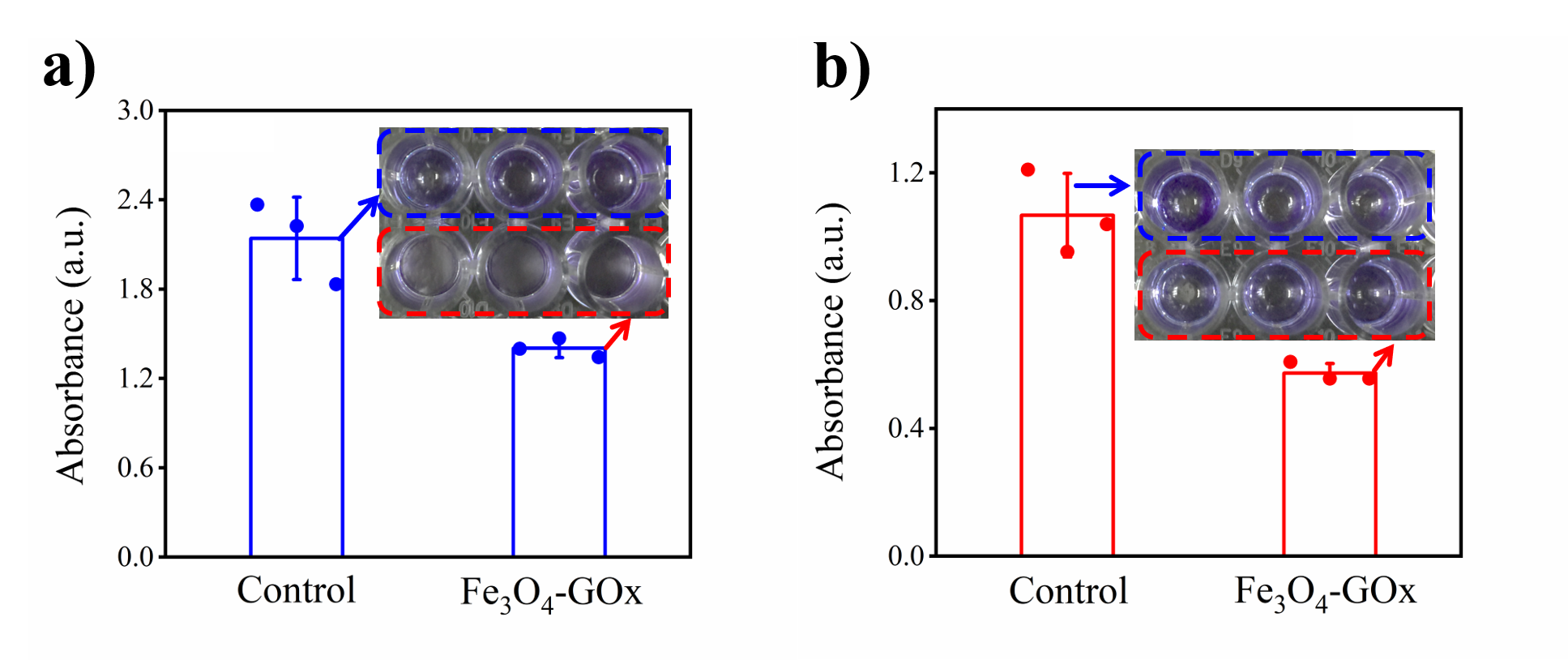
**

**Fig. S19** Crystal violet staining image and its corresponding absorbance for the formation of MRSA biofilm (a) and *E. coli* biofilm (b) in the presence of Fe_3_O_4_-GOx/glucose and control (PBS/glucose), respectively. The values of crystal violet absorbance represent the mean of three independent experiments, and the error bars indicate the SD from the mean.


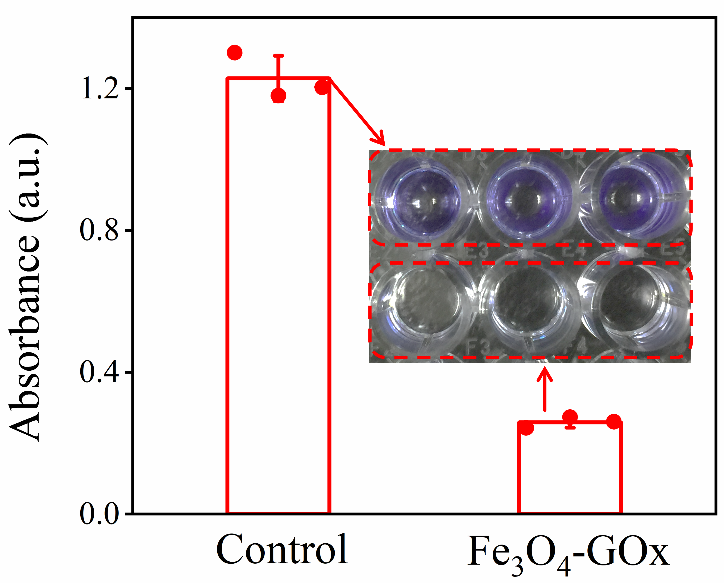


**Fig. S20**  Crystal violet staining image and its corresponding absorbance for integrated *E. coli* biofilm treated by the system of Fe_3_O_4_-GOx/glucose and control (PBS)/glucose, respectively. The inset shows the corresponding photographs of crystal violet staining of *E. coli* biofilm in the two treatment groups. The values of crystal violet absorbance represent the mean of three independent experiments, and the error bars indicate the SD from the mean.


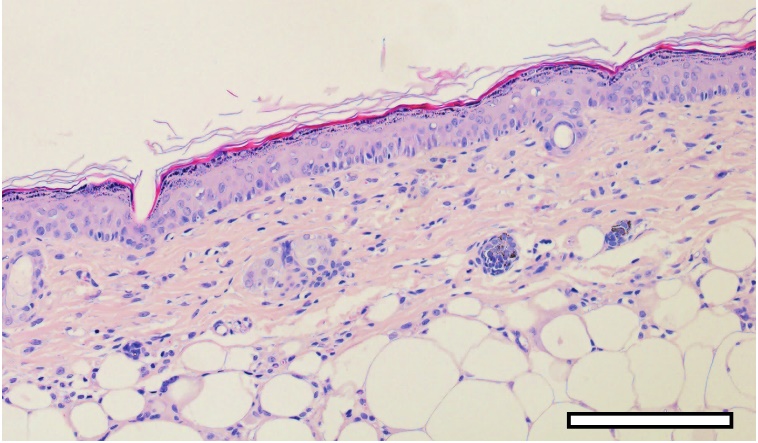


**Fig. S21** Representative histological HE staining images of normal skin tissue of diabetic mice. Scale bar: 150 µm.


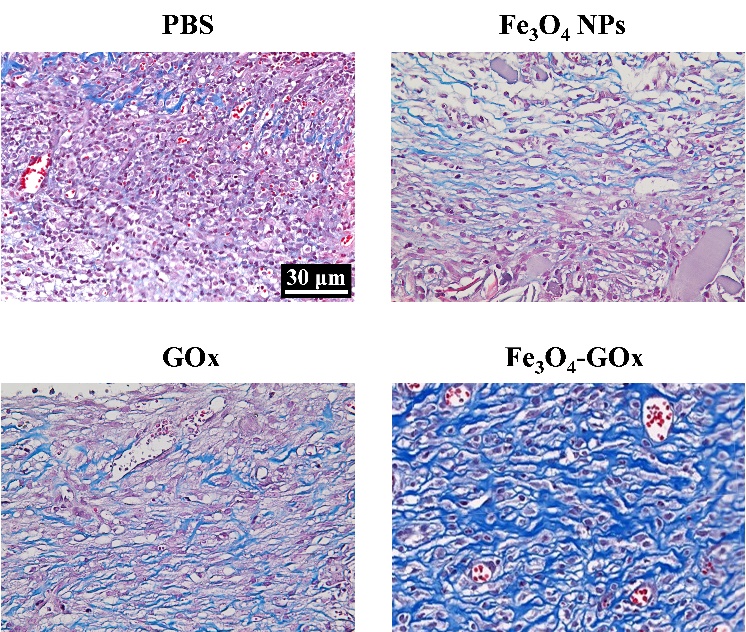


**Fig. S22** Magnified Masson’s trichrome staining images shown in Figure 6a (red rectangles)

indicate the condition of fiber alignment in four treatment groups.


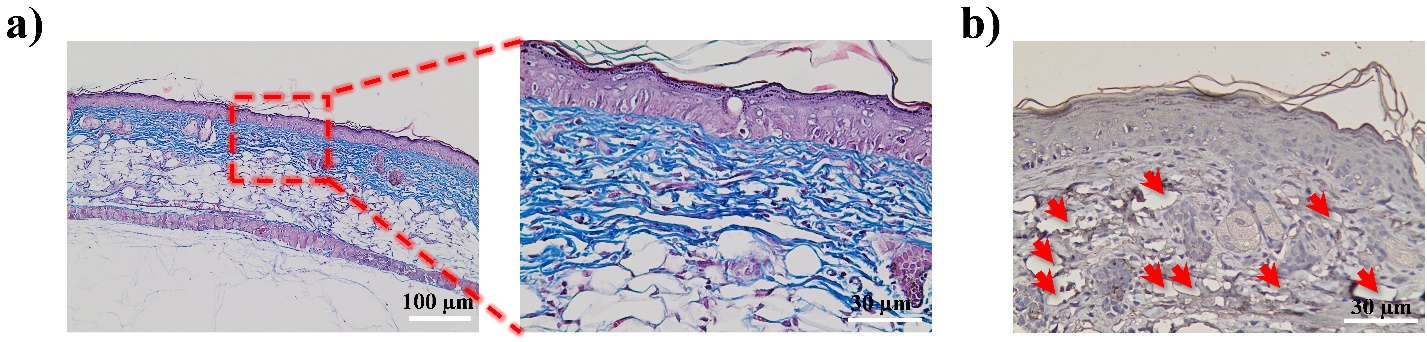


**Fig. S23** Representative Masson’s trichrome staining (a) and representative CD31 staining (b) images of normal skin tissue of diabetic mice.

**
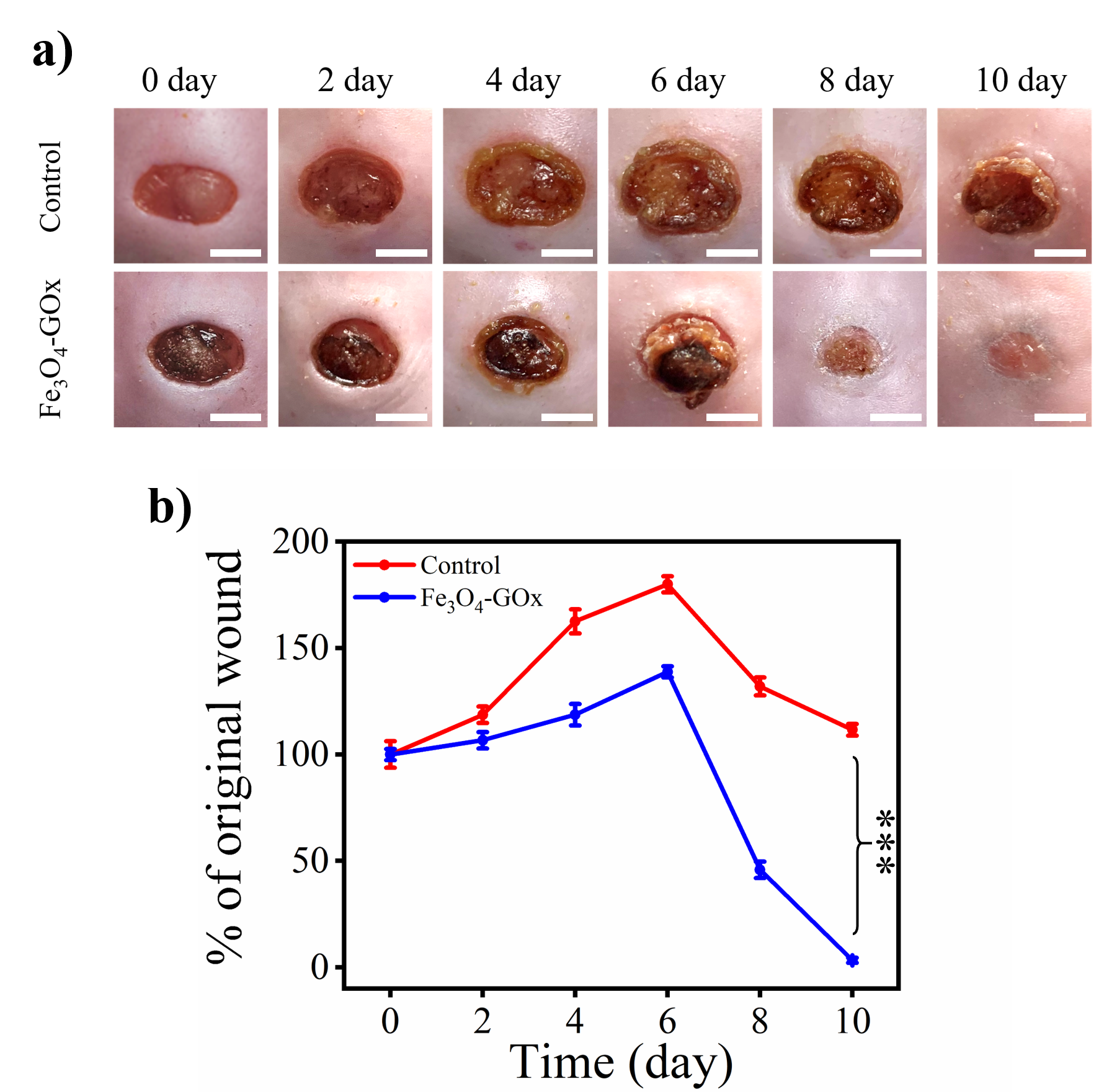
**

**Fig. S24**  (a) Representative photographs of non-infected diabetic wound in the treatment groups of control (PBS) and Fe_3_O_4_-GOx within 10 days. Scale bar: 5 mm. (b) Corresponding values of wound area in the two treatment groups shown in (a). The values of wound healing ratios (% of original wound) represent the mean of three independent experiments, and the error bars indicate the SD from the mean. ****P* < 0.001.

**
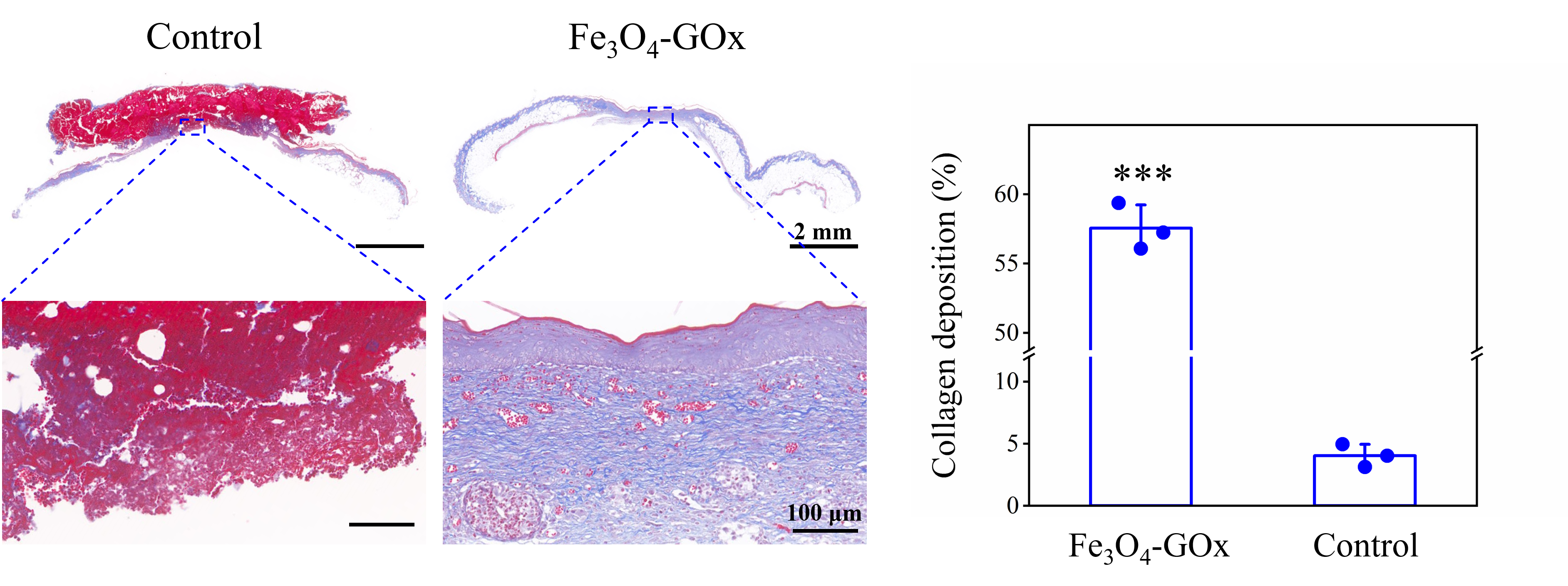
**

**Fig. S25** Representative Masson’s trichrome staining images and the corresponding quantitative data of collagen deposition percentage of the tissues of non-infected diabetic wound after 10 days treatment of control (PBS) and Fe_3_O_4_-GOx, respectively. The values of collagen deposition percentage represent the mean of three independent experiments, and are stated as mean ± SD. ****P* < 0.001.

**
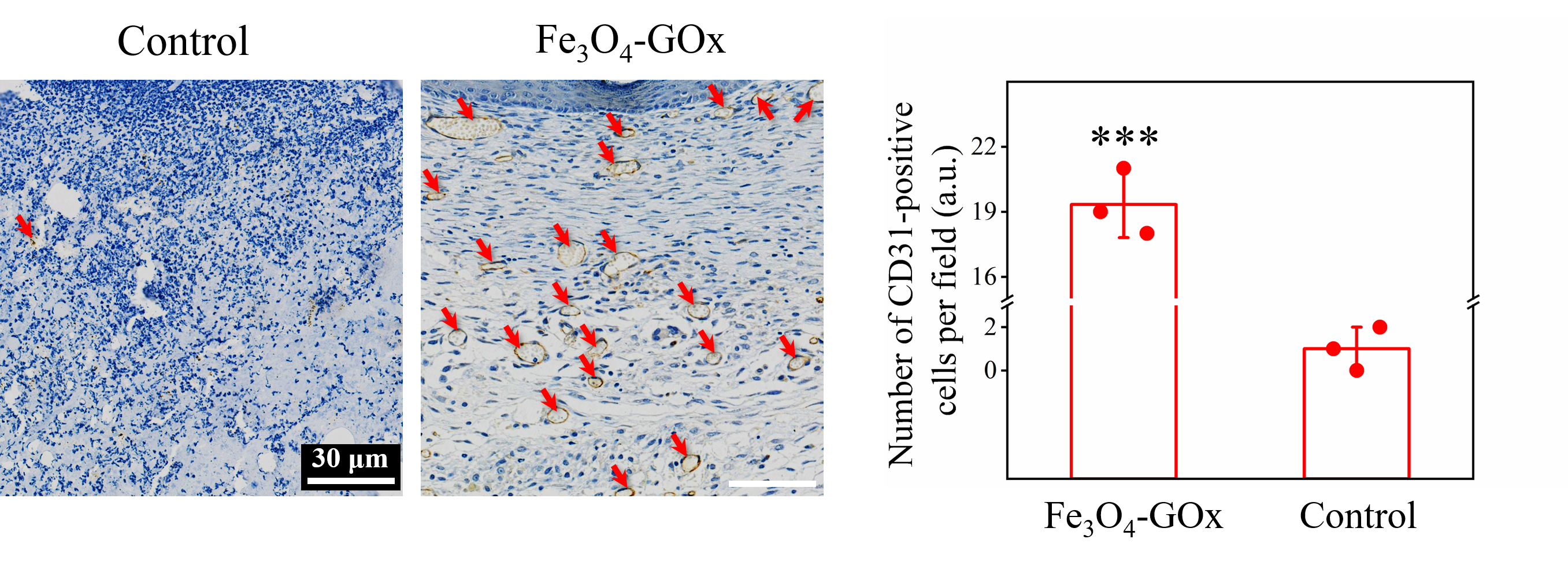
**

**Fig. S26** Representative CD31 staining images and the corresponding quantitative data of the CD31-positive cell’s number of the tissues of non-infected diabetic wound after 10 days treatment of control (PBS) and Fe_3_O_4_-GOx, respectively. The values of CD31-positive cell’s number represent the mean of three independent experiments, and are stated as mean ± SD. ****P* < 0.001.

**
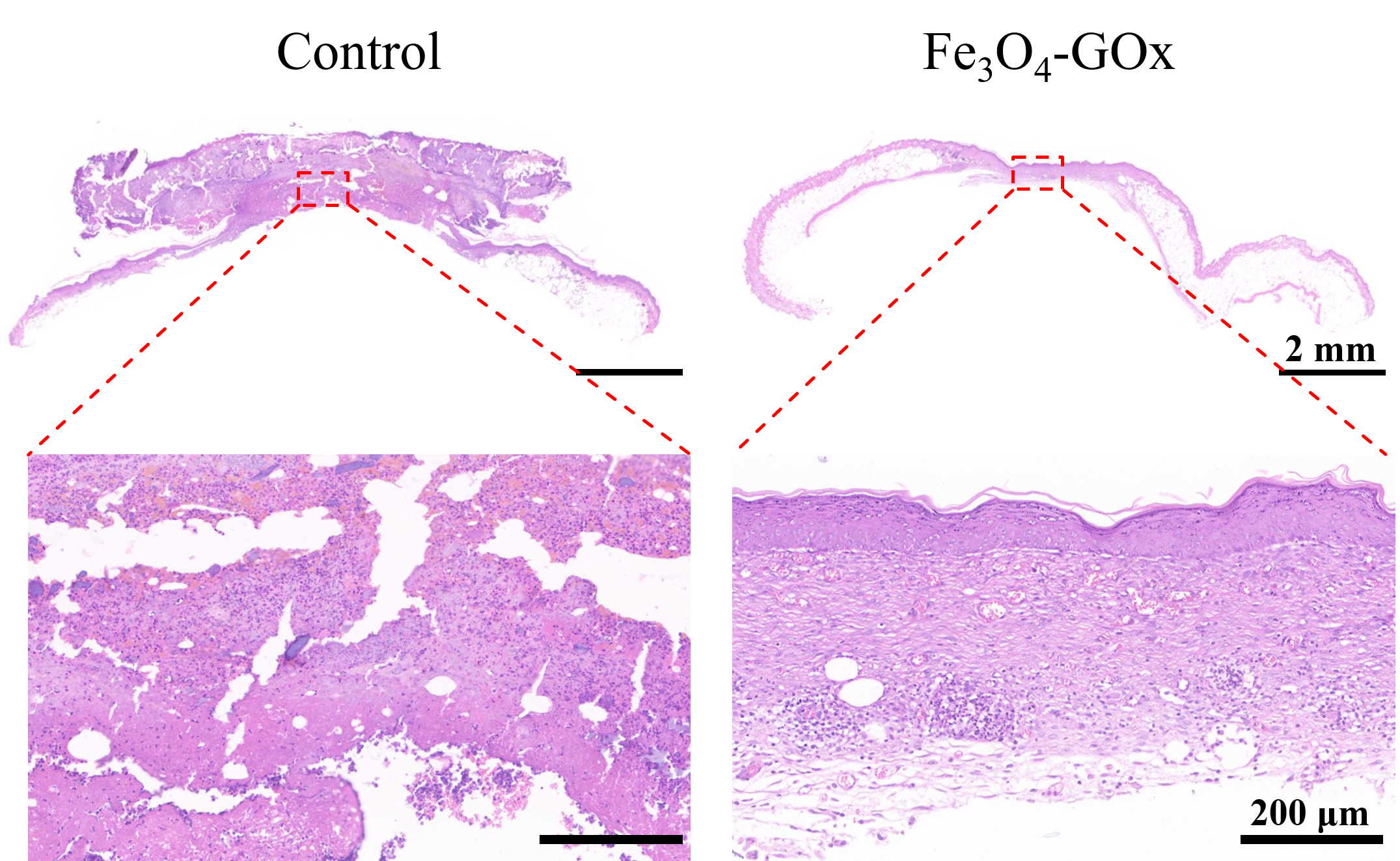
**

**Fig. S27** Representative hematoxylin and eosin (HE) staining images of non-infected diabetic wound after 10 days treatment of PBS and Fe_3_O_4_-GOx, respectively.





**Fig. S28** Proliferation of HUVEC cells after incubation with Fe_3_O_4_-GOx, Fe_3_O_4_ NPs, and GOx respectively for different times. The values of cell viability represent the mean of three independent experiments, and the error bars indicate the SD from the mean.


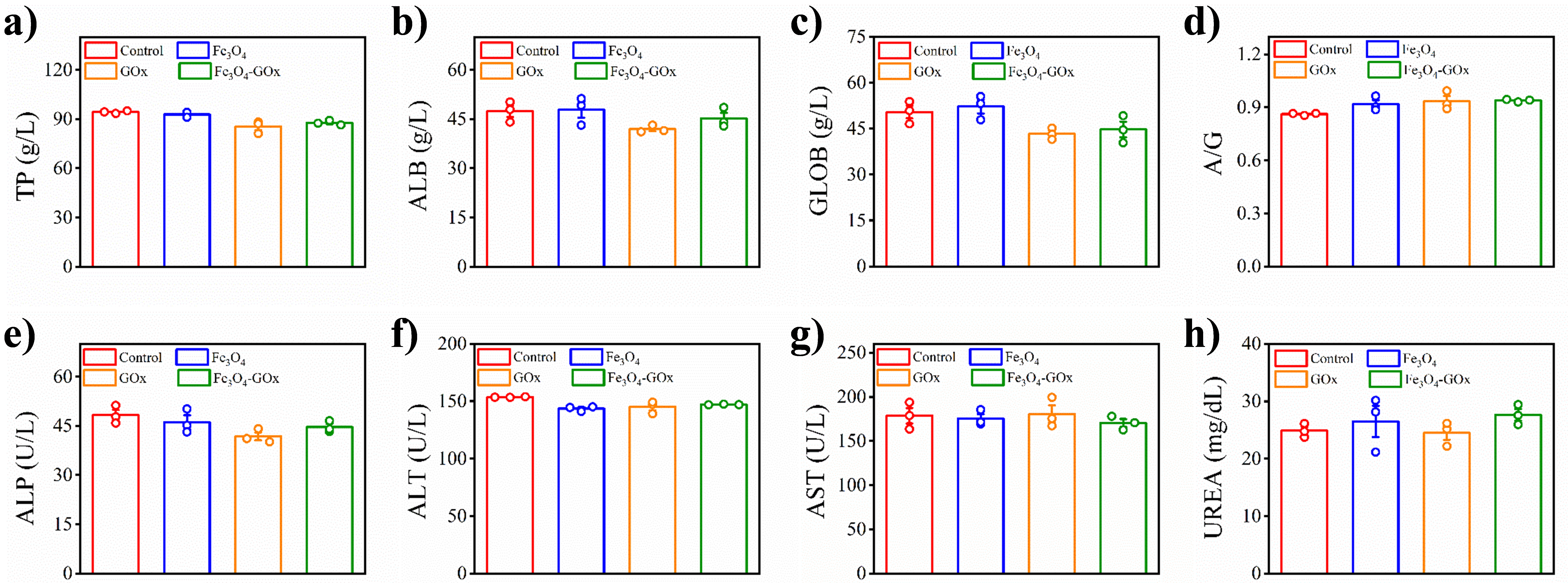


**Fig. S29** Blood biochemistry data of diabetic mice treated with Fe_3_O_4_-GOx, Fe_3_O_4_ NPs, GOx, and control (PBS) for 7 days. In these data, TP stands for total protein, GLOB stands for globulin, ALB stands for albumin, A/G stands for the ratio of albumin to globulin, ALP stands for alkaline phosphatase, ALT stands for alanine aminotransferase, AST stands for aspartate aminotransferase, and UREA stands for urea nitrogen.


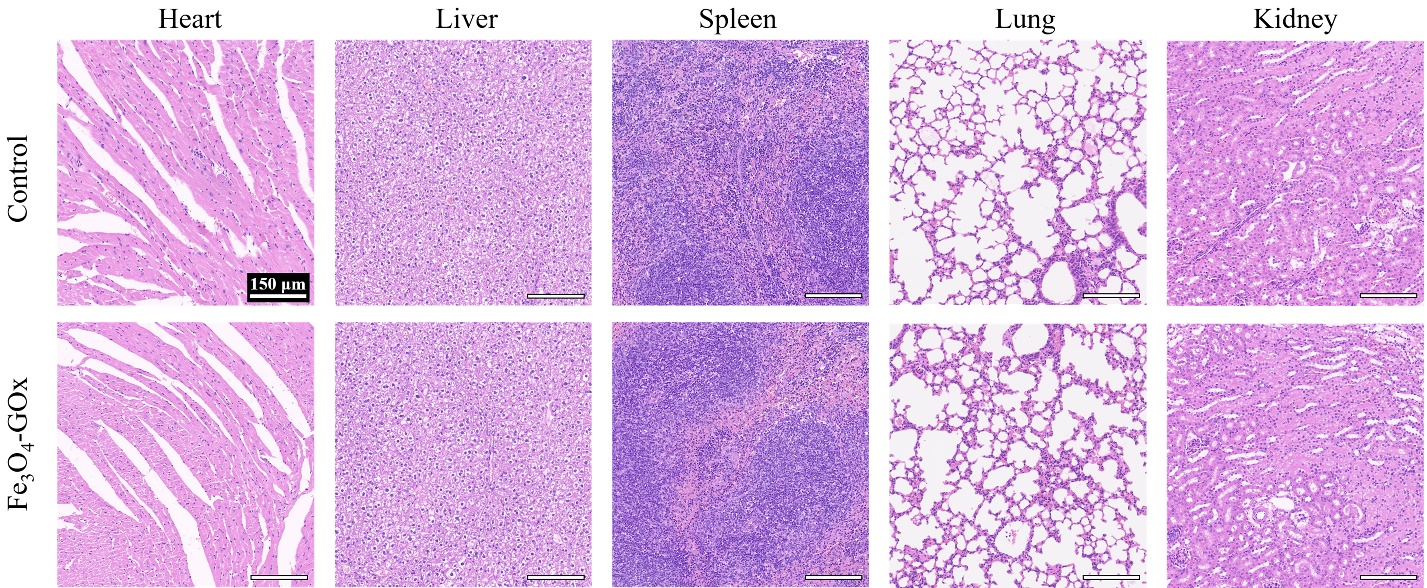


**Fig. S30** Representative H&E staining images of major organs (heart, liver, spleen, lung and kidney) of diabetic mice treated with Fe_3_O_4_-GOx and control (PBS) for 7 days.
